# Artificial Intelligence for Screening of Multiple Retinal and Optic Nerve Diseases

**DOI:** 10.1001/jamanetworkopen.2022.9960

**Published:** 2022-05-03

**Authors:** Li Dong, Wanji He, Ruiheng Zhang, Zongyuan Ge, Ya Xing Wang, Jinqiong Zhou, Jie Xu, Lei Shao, Qian Wang, Yanni Yan, Ying Xie, Lijian Fang, Haiwei Wang, Yenan Wang, Xiaobo Zhu, Jinyuan Wang, Chuan Zhang, Heng Wang, Yining Wang, Rongtian Chen, Qianqian Wan, Jingyan Yang, Wenda Zhou, Heyan Li, Xuan Yao, Zhiwen Yang, Jianhao Xiong, Xin Wang, Yelin Huang, Yuzhong Chen, Zhaohui Wang, Ce Rong, Jianxiong Gao, Huiliang Zhang, Shouling Wu, Jost B. Jonas, Wen Bin Wei

**Affiliations:** 1 Beijing Tongren Eye Center, Beijing Key Laboratory of Intraocular Tumor Diagnosis and Treatment, Beijing Ophthalmology and Visual Sciences Key Lab, Medical Artificial Intelligence Research and Verification Key Laboratory of the Ministry of Industry and Information Technology, Beijing Tongren Hospital, Capital Medical University, Beijing, China; 2 Beijing Airdoc Technology Co, Ltd, Beijing, China; 3 eResearch Centre, Monash University, Melbourne, Victoria, Australia; 4 ECSE, Faculty of Engineering, Monash University, Melbourne, Victoria, Australia; 5 Beijing Institute of Ophthalmology, Beijing Ophthalmology and Visual Science Key Lab, Beijing Tongren Eye Center, Beijing Tongren Hospital, Capital Medical University, Beijing, China; 6 Department of Ophthalmology, Shanxi Provincial People's Hospital, Taiyuan, China; 7 Department of Ophthalmology, Beijing Liangxiang Hospital, Capital Medical University, Beijing, China; 8 Department of Ophthalmology, Fuxing Hospital, Capital Medical University, Beijing, China; 9 Department of Ophthalmology, Xuanwu Hospital, Capital Medical University, Beijing, China; 10 Department of Ophthalmology, Dongfang Hospital, Beijing University of Chinese Medicine, Beijing, China; 11 Department of Ophthalmology, the Second Affiliated Hospital of Anhui Medical University, Hefei, China; 12 iKang Guobin Healthcare Group Co, Ltd, Beijing, China; 13 Department of Cardiology, Kailuan General Hospital, Tangshan, Hebei, China; 14 Department of Ophthalmology, Medical Faculty Mannheim, Heidelberg University, Mannheim, Germany; 15 Institute of Molecular and Clinical Ophthalmology Basel, Switzerland

## Abstract

**Importance:**

The lack of experienced ophthalmologists limits the early diagnosis of retinal diseases. Artificial intelligence can be an efficient real-time way for screening retinal diseases.

**Objective:**

To develop and prospectively validate a deep learning (DL) algorithm that, based on ocular fundus images, recognizes numerous retinal diseases simultaneously in clinical practice.

**Design, Setting, and Participants:**

This multicenter, diagnostic study at 65 public medical screening centers and hospitals in 19 Chinese provinces included individuals attending annual routine medical examinations and participants of population-based and community-based studies.

**Exposures:**

Based on 120 002 ocular fundus photographs, the Retinal Artificial Intelligence Diagnosis System (RAIDS) was developed to identify 10 retinal diseases. RAIDS was validated in a prospective collected data set, and the performance between RAIDS and ophthalmologists was compared in the data sets of the population-based Beijing Eye Study and the community-based Kailuan Eye Study.

**Main Outcomes and Measures:**

The performance of each classifier included sensitivity, specificity, accuracy, F1 score, and Cohen κ score.

**Results:**

In the prospective validation data set of 208 758 images collected from 110 784 individuals (median [range] age, 42 [8-87] years; 115 443 [55.3%] female), RAIDS achieved a sensitivity of 89.8% (95% CI, 89.5%-90.1%) to detect any of 10 retinal diseases. RAIDS differentiated 10 retinal diseases with accuracies ranging from 95.3% to 99.9%, without marked differences between medical screening centers and geographical regions in China. Compared with retinal specialists, RAIDS achieved a higher sensitivity for detection of any retinal abnormality (RAIDS, 91.7% [95% CI, 90.6%-92.8%]; certified ophthalmologists, 83.7% [95% CI, 82.1%-85.1%]; junior retinal specialists, 86.4% [95% CI, 84.9%-87.7%]; and senior retinal specialists, 88.5% [95% CI, 87.1%-89.8%]). RAIDS reached a superior or similar diagnostic sensitivity compared with senior retinal specialists in the detection of 7 of 10 retinal diseases (ie, referral diabetic retinopathy, referral possible glaucoma, macular hole, epiretinal macular membrane, hypertensive retinopathy, myelinated fibers, and retinitis pigmentosa). It achieved a performance comparable with the performance by certified ophthalmologists in 2 diseases (ie, age-related macular degeneration and retinal vein occlusion). Compared with ophthalmologists, RAIDS needed 96% to 97% less time for the image assessment.

**Conclusions and Relevance:**

In this diagnostic study, the DL system was associated with accurately distinguishing 10 retinal diseases in real time. This technology may help overcome the lack of experienced ophthalmologists in underdeveloped areas.

## Key Points

**Question:**

Can deep learning (DL) algorithms that recognize multiple retinal diseases simultaneously be applied in a clinical setting?

**Findings:**

In this diagnostic study, a DL system achieved 89.8% sensitivity to detect any of 10 retinal abnormalities. Compared with senior retinal specialists, the DL system reached a superior or similar diagnostic sensitivity in detecting 7 of 10 retinal diseases (ie, referral diabetic retinopathy, referral possible glaucoma, macular hole, epiretinal macular membrane, hypertensive retinopathy, myelinated fibers, and retinitis pigmentosa).

**Meaning:**

These findings suggest that a DL system can accurately distinguish 10 retinal diseases in real time and may help overcome the lack of experienced ophthalmologists in underdeveloped areas.

## Teaser

This diagnostic study develops and prospectively validates a deep learning algorithm that uses ocular fundus images to recognize numerous retinal diseases in a clinical setting at 65 screening centers in 19 Chinese provinces.

## Introduction

Retinal and optic nerve diseases have become the most common causes for irreversible vision loss globally.^1^ Their diagnosis relies on the availability of experienced ophthalmologists, however, the density of ophthalmologists varies within countries and regions. For example, there are approximately 40 000 registered ophthalmologists in China with a higher density in east China than in west China.^2^ In addition, the clinical experience of ophthalmologists varies, which further increases the unbalanced distribution of personal resources in ophthalmology.^3^ These regional differences limit the possibility of people living in underdeveloped regions to have regular screening for retinal diseases.

Thus, there is an increasing interest in establishing reliable and cost-effective methods for screening retinal diseases.^4,5^ Deep learning (DL)-based screening and referral of patients may help to overcome the shortage of experienced ophthalmologists in underdeveloped regions. The applications of DL techniques trained on color fundus images have shown great potential to provide close to expert performance for the automatic detection of retinal diseases, including diabetic retinopathy (DR),^6,7,8^ age-related macular degeneration (AMD),^9,10,11^ glaucoma,^12,13,14^ myopic maculopathy,^15^ retinopathy of prematurity,^16,17,18^ and papilledema.^19^ Although these systems have achieved a good diagnostic performance, nearly all of them could detect only 1 disease or a few diseases and lacked a prospective validation in a clinical setting.^6,20^ As an attempt, Choi et al^21^ performed a pilot study to develop a DL-associated model to detect 9 retinal diseases. With a relatively small sample size, this model showed an overall accuracy of 30.5%. Subsequent studies developed other algorithms for detection of several retinal diseases.^22,23,24,25,26,27^ Recently, Lin et al^27^ developed an algorithm that could detect 14 retinal abnormalities, with a mean sensitivity and specificity of 0.907 and 0.903, respectively. However, the model was only prospectively validated in 18 163 images.

In this study, we developed a DL algorithm, Retinal Artificial Intelligence Diagnosis System (RAIDS), to detect 10 retinal diseases simultaneously. We applied and tested RAIDS at 65 screening centers in 19 provinces of China and compared its performance with the results of a reader study including retinal experts.

## Methods

### Study Design and Overview

This diagnostic study was approved by the Medical Ethics Committee of Beijing Tongren Hospital and the Ethics Committee of the iKang Corporation. All fundus images were deidentified before the analysis. Written informed consent was obtained in the retrospective data set. In the prospective data set, oral informed consent was obtained from all participants, while written informed consent was exempted by the Ethics Committee of the iKang Corporation. This study followed the Standards for Reporting of Diagnostic Accuracy (STARD) reporting guideline.

We first developed RAIDS to detect multiple retinal diseases from fundus images using a retrospective data set. The system was then tested in a prospective data set in a clinical setting. RAIDS was further validated in a reader study to compare the performance and efficiency with human ophthalmologists. The study was registered in ClinicalTrials.gov (NCT04678375 and NCT04592068).

### Development of RAIDS

For the developmental data set, fundus images were retrospectively collected in 10 iKang Health Care centers and the Beijing Tongren Hospital between June 2018 and June 2020. All images came from different eyes, and the demographic information of each image was masked during the analysis. In a first round of the assessment, the fundus images were examined by 3 randomly chosen examiners of a group of 40 certified ophthalmologists. The diagnosis was final if at least 2 examiners agreed in their diagnosis, otherwise, the image was reassessed in a second round by a panel of 6 senior experts. This development data set was randomly split into a training data set and a testing data set with a ratio of 5 to 1 (eTable 1 in the Supplement). The overall architecture of the multitask convolutional neural network (CNN) included a first part containing a model for the macula and optic disc region and a second part consisting of a model for multitask learning for the retinal disease classification.

In a first step, the retinal disorders were divided into 3 groups: the general group (ie, DR, pathologic myopia, retinal vein occlusions, hypertensive retinopathy, myelinated fibers, and retinitis pigmentosa), the macula group (ie, AMD, macula hole and epiretinal macular membrane), and the optic disc group (ie, glaucoma). Therefore, we designed a multitask classification framework with 3 subtasks: the general subtask for all the 10 kinds of abnormalities, the macula subtask for the macula abnormalities, and the optic-disc subtask for the optic-disc abnormalities.

In a second step, a joint CNN detector using Yolov3 was trained to localize the regions of the macula and optic nerve head.^28^ The macula and optic nerve head regions were then generated by extending the originally detected bounding boxes by one-fourth of the widths and heights to cover the neighboring regions of the macula and optic nerve head. After detection, each subtask took the corresponding interesting region of the fundus image as the input. In addition to the classes of retinal abnormalities, each subtask contained a healthy class describing the absence of retinal diseases. During inference, each subtask reported the status as either healthy or abnormal with some retinal diseases. An eye was diagnosed as healthy if none of the 3 subtasks indicated abnormal, otherwise, the multitask classification output was the union of the reported abnormalities of the 3 subtasks.

### Prospective Dataset Validation in a Clinical Setting

To validate the applicability of the RAIDS system in a screening scenario in a clinical setting, a prospective investigation was conducted in cooperation with 65 examination centers of the iKang Guobin Health Physical Examination Management Group Co, which were different from the 10 examination centers involved for the creation of the development data set. The 65 private examination centers where the participants underwent annual eye examinations were located in cities across 19 Chinese provinces. The examinations were carried out from November 2020 to February 2021. In each center, the RAIDS was deployed in the cloud, which was connected to the fundus cameras through the internet. Using nonmydriatic 45-degree fundus cameras, the study participants underwent fundus photography performed by trained operators. The operators identified images nonassessable for a correct diagnosis of fundus abnormalities, because of reasons such as blur and defocus, and excluded them from further analysis. The remaining fundus images were saved in the jpeg format and uploaded to the online database, when the RAIDS performed a first analysis in real time. Within 8 hours, 2 experts from the expert panel consisting of 45 certified retinal specialists with more than 5 years of clinical experience prepared a medical report for each image. The 2 certified experts separately examined each image, and a conclusion was drawn in the case of an agreement of both experts. Otherwise, an arbitration was performed by a third specialist. The managers in the health care centers then informed the participants of the medical report made by the experts.

### AI–Human Ophthalmologist Comparison

Two population-based studies, the Beijing Eye Study^29^ and the Kailuan Eye Study,^30^ were used as external validation data sets. In brief, the Beijing Eye Study was conducted in 2011 in 7 communities, 3 of which were located in rural regions and 4 of which were located in urban regions of Beijing, China. The Kailuan Eye Study was performed in 2014 and included employees and retirees of a coal mining company in Tangshan, China. All study participants underwent fundus photography (fundus camera CR6-45NM, Canon). The expert panel consisted of 6 retinal experts with more than 15 years of experience to annotate the images. Each image was randomly assigned to 2 experts to generate the reference standard. The image labeling was finalized only when both retinal experts reached a consensus, otherwise, the final decision would be made by a third expert.

To compare the performance between the RAIDS and ophthalmologists, three doctor groups including 9 ophthalmologists were asked to independently diagnose the same images in the reader study: 3 senior retinal specialists (10 to 15 years of experience with retinal diseases), 3 junior retinal specialists (5 to 10 years of experience with retinal diseases), and 3 certified ophthalmologists (less than 5 years of experience with retinal diseases).

### Statistical Analysis

Because the demographic parameter of age was not normally distributed, it was described by its median and range or IQR. To visualize the decision ways of the model, we applied the Grad-CAM to generate heatmaps.^31^ The statistical analyses were performed using R version 4.0.3, (R Project for Statistical Computing). The performance of RAIDS was described by its accuracy, sensitivity, and specificity to identify each of the diseases. We additionally assessed the F1 score and Cohen κ score. An assessment of a receiver operating characteristic curve and of the area under curve (AUC) was not applicable for the multitask framework. N-out-of-N bootstrapping was used to estimate the 95% CIs of the performance metrics at the image level. The bootstrap sampling was repeated 2000 times. The details of the metric evaluation were shown in eMethods in the Supplement.

## Results

### Development of RAIDS

To develop RAIDS, the study used 120 002 fundus images taken from 63 400 individuals (34 030 [53.7%] women) with a median (IQR) age of 44 (32-55) years with a range of 9 to 85 years. In the internal test data set, RAIDS exhibited an accuracy of 83.0% in classifying normal from all fundus images and could distinguish 89.3% of abnormal images of any kind. Details of the performance of RAIDS in the 10 diseases are listed in eTable 2 in the Supplement.

### Prospective Validation in Screening Setting

**Figure 1.**
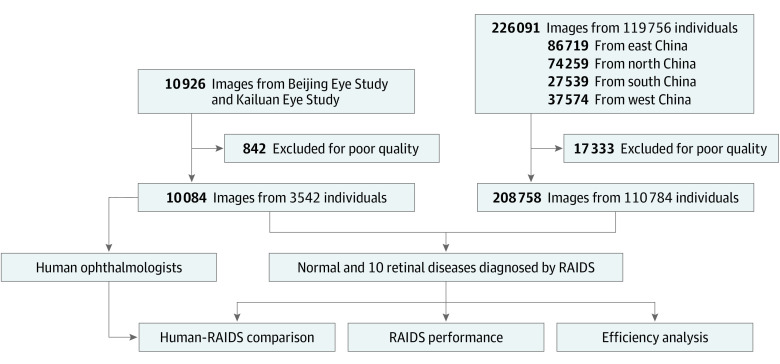
Flow Chart of the Included Participants in Prospective Validation Study and Reader Study RAIDS indicates Retinal Artificial Intelligence Diagnosis System.

From November 2020 to February 2021, a total of 226 091 images from 119 756 participants (median [range] age, 41 [8-87] years) were collected from 65 health care centers located throughout China (Figure 1). Of these images, 17 333 were excluded because they were not of clinically acceptable quality, and a total of 208 758 images from 110 784 participants were included in the study (Table; eFigure 1 and eFigure 2 in the Supplement). Among the included individuals, the median (range) age was 42 (8-87) years, 115 443 participants (55.3%) were female, and 170 038 images (81.5%) were labeled as normal.

**Table.** Baseline Characteristics of Participants and Images in Prospective Screening Dataset

Characteristics	Participants, No. (%)				
	East China	North China	South China	West China	Total
Participants, No.	42 069	36 685	13 616	18 414	110 784
Age, median (range), y	43 (8-87)	42 (9-87)	39 (13-85)	42 (8-85)	42 (8-87)
Female	23 834 (56.7)	19 647 (53.6)	7129 (52.4)	10 728 (58.3)	61 338 (55.4)
Male	18 235 (43.3)	17 038 (46.4)	6487 (47.6)	7686 (41.7)	49 446 (44.6)
Images, No.	79 453	69 392	25 366	34 517	208 758
Normal	64 948 (81.7)	55 463 (79.9)	21 984 (86.7)	27 643 (80.1)	170 038 (81.5)
Referral					
DR	2046 (2.6)	2642 (3.8)	500 (2.0)	1320 (3.8)	6508 (3.1)
AMD	2851 (3.6)	2308 (3.3)	708 (2.8)	1166 (3.4)	7033 (3.4)
Possible glaucoma	3871 (4.9)	3352 (4.8)	1142 (4.5)	1849 (5.4)	10214 (4.9)
Pathological myopia	1000 (1.3)	449 (0.6)	185 (0.7)	471 (1.4)	2105 (1.0)
Retinal vein occlusion	257 (0.3)	264 (0.4)	43 (0.2)	139 (0.4)	703 (0.3)
Macula hole	78 (0.1)	92 (0.1)	25 (0.1)	40 (0.1)	235 (0.1)
Epiretinal macular membrane	1525 (1.9)	1380 (2.0)	445 (1.8)	681 (2.0)	4031 (1.9)
Hypertensive retinopathy	2825 (3.6)	3487 (5.0)	534 (2.1)	1352 (3.9)	8198 (3.9)
Myelinated fibers	321 (0.4)	241 (0.3)	89 (0.4)	161 (0.5)	812 (0.4)
Retinitis pigmentosa	34 (0)	16 (0)	13 (0.1)	27 (0.1)	90 (0)

Abbreviations: AMD, age-related macular degeneration; DR, diabetic retinopathy.

**Figure 2.**
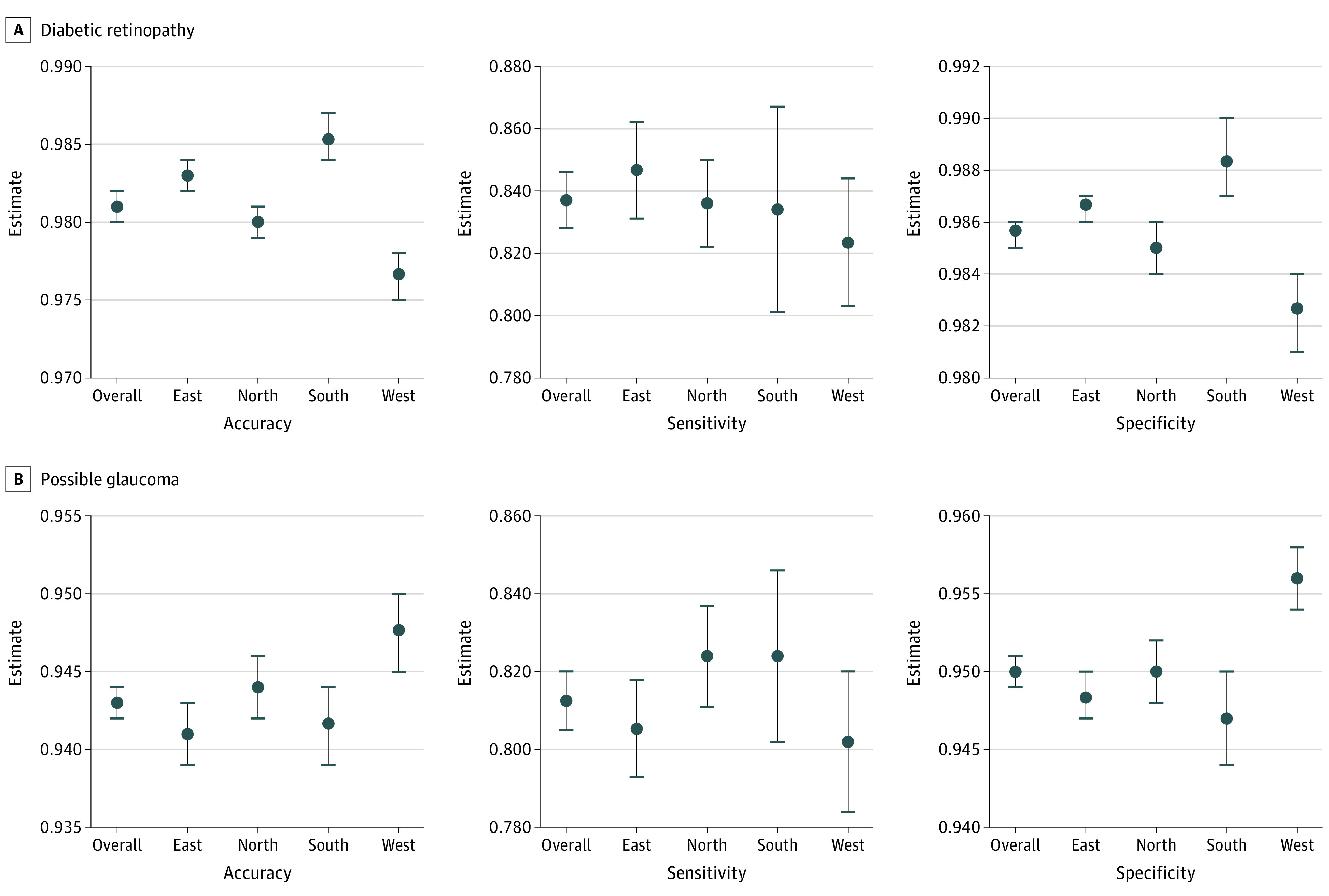
Performance of the RAIDS in Prospective Validation Dataset RAIDS indicates Retinal Artificial Intelligence Diagnosis System.

The sensitivity, specificity, and F1 score of RAIDS for identifying normal retinal images reached 0.780 (95% CI, 0.778-0.782), 0.898 (95% CI, 0.895-0.901), and 0.865 (95% CI, 0.864-0.866), respectively. Of the included images, 38 720 (18.5%) were diagnosed as abnormal. RAIDS accurately identified the accuracy (95% CI) of referral DR (0.981 [0.980-0.982]), referral AMD (0.973 [0.972-0.973]), referral possible glaucoma (0.943 [0.942-0.944]), pathological myopia (0.984 [0.983-0.984]), retinal vein occlusion (0.974 [0.973-0.975]), macular hole (0.996 [0.995-0.996]), epiretinal macular membrane (0.978 [0.977-0.979]), hypertensive retinopathy (0.837 [0.836-0.839]), myelinated fibers (0.999 [0.999-0.999]), and retinitis pigmentosa (0.999 [0.999-1.000]) (Figure 2; eFigure 3 and eTable 3 in the Supplement). Differentiating between the various regions of China, the sensitivity of RAIDS for detecting any retinal disorder ranged from 90.7% to 92.0% (east China: 0.915 [0.911-0.920]; north China: 0.920 [0.916-0.925]; south China: 0.907 [0.897-0.917]; west China: 0.915 [0.908-0.922] (Figure 2; eFigure 3 and eTables 3-7 in the Supplement).

### Heatmap Visualization

The heatmap analysis revealed that RAIDS preferentially assessed the macular region and the region of the optic nerve head (eFigures 4-13 in the Supplement). The regions of interest matched with lesions that ophthalmologists would pay attention to when making the diagnosis.

### AI–Human Ophthalmologist Comparison

From the Beijing Eye Study and the Kailuan Eye Study, 10 084 fundus images were used to compare the performance of RAIDS and human ophthalmologists (eTable 8 in the Supplement). RAIDS reached an accuracy of 0.953 (95% CI, 0.949-0.957), a sensitivity of 0.964 (95% CI, 0.960-0.968), and a specificity of 0.917 (95% CI, 0.906-0.928) in identifying normal from abnormal images (eTable 9 in the Supplement). RAIDS showed a higher specificity in identifying normal images than certified ophthalmologists (0.837 [95% CI, 0.821-0.851]), junior retinal specialists (0.864 [95% CI, 0.849-0.877]), and senior retinal specialists (0.885 [95% CI, 0.871-0.898]).

**Figure 3.**
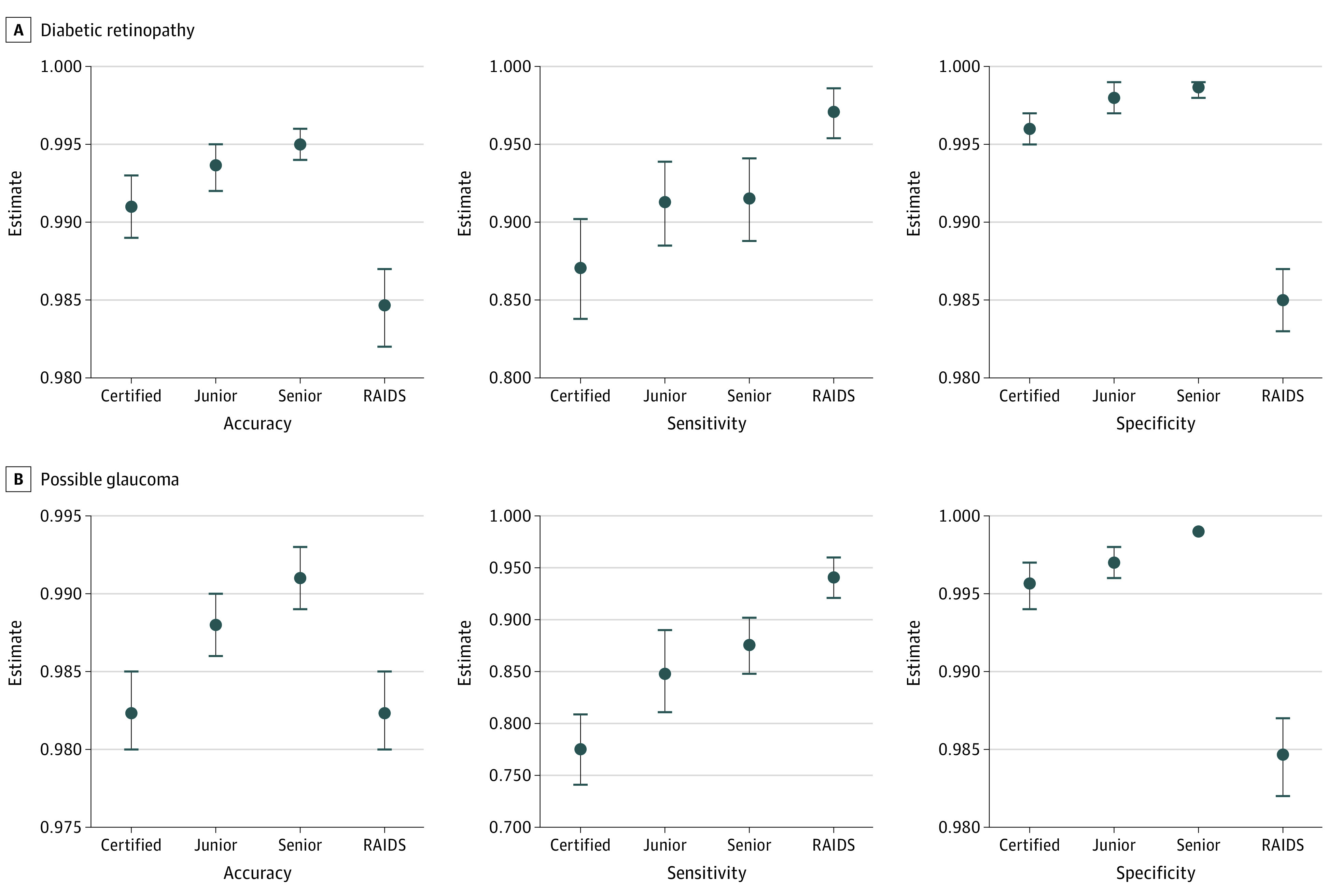
Accuracy, Sensitivity, and Specificity of Assessments of Ocular Fundus Photographs Performed by Certified Ophthalmologists, Junior Retinal Specialists, Senior Retinal Specialists, and by the Retinal Artificial Intelligence Diagnosis System Error bars indicate 95% CI; RAIDS, Retinal Artificial Intelligence Diagnosis System.

For diagnosing 10 retinal diseases, the accuracy of RAIDS ranged from 0.982 to 1.000, which was higher than or equal to the values reached by senior retinal specialists in 7 of 10 retinal diseases (AMD, retinal vein occlusions, macular holes, epiretinal macular membrane, hypertensive retinopathy, myelinated fibers, and retinitis pigmentosa) (Figure 3; eFigure 14 in the Supplement). RAIDS reached a superior or noninferior sensitivity as compared with retinal specialists in 7 of 10 retinal diseases (referral DR, referral possible glaucoma, macular hole, epiretinal macular membrane, hypertensive retinopathy, myelinated fibers, and retinitis pigmentosa). RAIDS showed an inferior diagnostic sensitivity as compared with ophthalmologists only for the detection of pathological myopia.

The mean κ score between RAIDS and the different groups of clinical experts ranged from 0.720 to 0.757, with RAIDS showed a higher κ score as compared with experienced experts (eTable 10 in the Supplement). The κ scores between junior and senior retinal specialists reached 0.912, while it was 0.769 between certified ophthalmologists and senior retinal specialists (eTable 11 in the Supplement).

The assessment of each image by the RAIDS, certified ophthalmologists, junior retinal specialists, and retinal specialists took about 0.30, 10.88, 8.68, and 7.68 seconds, respectively. The RAIDS alone could save 96% to 97% of the time needed by an ophthalmologist (eTable 12 in the Supplement).

Combining ophthalmologists and RAIDS in attempt to save time and achieve a better performance, we set a scenario where images would be regarded as normal if the RAIDS classified them as normal. Images detected to be abnormal by RAIDS would undergo the definite diagnosis by ophthalmologists. This combination reached a noninferior accuracy, a higher specificity, and a lower sensitivity as compared with using opthamologists or RAIDS alone (eFigure 15 and eTables 13-15 in the Supplement). The combination strategy needed about 75% less time as compared with ophthalmologists alone (eTable 12 in the Supplement).

## Discussion

In this multicenter diagnostic study conducted at public screening centers and hospitals throughout China, RAIDS achieved high diagnostic accuracy and sensitivity in detecting multiple retinal diseases and saved more than 95% of the examination time. Combining RAIDS with the clinical diagnosis by ophthalmologists achieved a similar diagnostic accuracy and reduced the time needed for examination by 75% as compared with an examination based on ophthalmologists alone.

The scarcity of ophthalmologists in rural regions and the lack of experts in opthamology are major factors limiting screening projects for the early detection of blinding diseases. Correspondingly, the agreement between the diagnoses made by certified ophthalmologists and by junior or senior retinal specialists was only moderate in our study. DL-based screening and referral systems may overcome these limitations.^32^ Previous studies aimed to discriminate several retinal diseases using a single algorithm.^22,23,24,25,26^ Using 4435 images from publicly available fundus image databases, Stevenson et al^33^ developed a DL model with a 6-categorical classification (normal retina and 5 retinal diseases). The mean diagnostic accuracy, sensitivity, specificity, and AUC was 89%, 75%, 89%, and 0.58, respectively. Son et al^24^ established DL models using larger data sets to detect 12 retinal abnormalities. The models achieved a similar diagnostic accuracy as compared with clinical experts, with a mean AUC higher than 95%. The study was limited because the system only detected abnormalities rather than retinal diseases, thus this model could not directly be used for screening. Recently, Lin et al^27^ developed a DL-based system that could detect 14 retinal diseases (ie, a higher number than the figure of 10 diseases assessed in our study). As an additional advantage, the Lin et al^27^ study population also included Asian patients, Black patients, Hispanic patients, and White patients. In contrast to our investigation, the Lin et al^27^ system was prospectively validated only in 18 163 images, and our study was more focused on application in a clinical setting. While both studies demonstrated the possibility of the systems developed to detect fundus abnormalities and diseases, RAIDS showed a higher sensitivity for the detection of 8 of all 8 comparable diseases, and a superior or noninferior specificity in 5 of all 8 comparable diseases. By using 2 population-based data sets (Beijing Eye Study and Kailuan Eye Study), the present investigation also included a reader study to evaluate the screening efficiency of RAIDS.^34^

In this study, RAIDS could distinguish 10 common retinal diseases with an accuracy ranging from 95.3% to 99.9%, without major differences between regions. The reader study revealed that the diagnostic accuracy of RAIDS was equal to or better than that of ophthalmologists, including experienced retinal specialists. Furthermore, RAIDS reached a superior or noninferior diagnostic sensitivity compared with retinal specialists in 7 of 10 retinal diseases. These data suggest that RAIDS could be used to provide an independent and automated feedback in health care centers and make referral suggestions. It might help eliminate the gap of resource distribution in underdeveloped regions. In the prospective validation study, all retinal images were evaluated by experts of a retinal expert panel, and referral suggestions were given by phone. The results of the prospective validation revealed that RAIDS had a performance comparable with the retinal experts and suggest that a referral mechanism may be established between local hospitals and medical examinations or reading centers equipped with RAIDS.

Besides an independent and automated feedback, RAIDS can also be used along with ophthalmologists, as also examined in studies by Li et al^26^ and Kim et al.^27^ In our study, the combination of RAIDS for the detection of a fundus abnormalities and ophthalmologists for the final diagnosis achieved a similar performance compared with ophthalmologists alone but saved close to 75% of the examination time.

### Limitations

This study had limitations. First, the RAIDS showed a lower diagnostic performance in the prospective data set than in the 2 population-based data sets. Second, the image quality control was performed by ophthalmologists, which may not be the case in a clinical setting. Third, because the number of images with retinitis pigmentosa was relatively small, the performance of RAIDS could not be validly tested for the detection of this disorder. A strength of the system was that the technology was agnostic to how the retinal images are taken so that costly, hard to disseminate technologies or platforms were not needed to implement the system in a clinical setting.

## Conclusions

These findings suggest that RAIDS achieved a high accuracy in detecting multiple retinal disorders and saved examination time for the screening. RAIDS might be used to provide an automated and immediate referral suggestion in screening and primary care clinic settings, particularly in undeveloped areas, to overcome the shortage of medical resources.

## Notes

Supplement.
eMethods.
**eFigure 1.** The Overall Structure of Study **eFigure 2.** Distribution of Images in Prospective Validation Dataset **eFigure 3.** Performance of the RAIDS in Prospective Validation Dataset **eFigure 4.** Heatmap Visualization of Referral Diabetic Retinopathy **eFigure 5. **Heatmap Visualization of Referral Age-Related Macular Degeneration **eFigure 6.** Heatmap Visualization of Referral Possible Glaucoma **eFigure 7.** Heatmap Visualization of Pathological Myopia **eFigure 8.** Heatmap Visualization of Retinal Vein Occlusion **eFigure 9.** Heatmap Visualization of Macula Hole **eFigure 10.** Heatmap Visualization of Epiretinal Macular Membrane **eFigure 11.** Heatmap Visualization of Hypertensive Retinopathy **eFigure 12.** Heatmap Visualization of Myelinated Fibers **eFigure 13.** Heatmap Visualization of Retinitis Pigmentosa **eFigure 14.** Performance Comparison Between RAIDS and Human Ophthalmologists in Identifying Multiple Retinal Diseases **eFigure 15.** Performance of RAIDS-Human Ophthalmologist Combination in Identifying Multiple Retinal Diseases **eTable 1.** Development Dataset Distributions of Retinal Diseases and Camera Manufacturers **eTable 2.** Performance of the Retinal Artificial Intelligence Diagnosis System in the Internal Validation Dataset **eTable 3.** Performance of the Retinal Artificial Intelligence Diagnosis System (RAIDS) in Prospective Validation Dataset **eTable 4.** Performance of the Retinal Artificial Intelligence Diagnosis System in Identifying Multiple Retinal Diseases in East China **eTable 5.** Performance of the Retinal Artificial Intelligence Diagnosis System in Identifying Multiple Retinal Diseases in North China **eTable 6.** Performance of the Retinal Artificial Intelligence Diagnosis System Identifying Multiple Retinal Diseases in South China **eTable 7.** Performance of the Retinal Artificial Intelligence Diagnosis System in Identifying Multiple Retinal Diseases in West China **eTable 8.** Baseline Characteristics of Participants in the Reader Study **eTable 9.** Performance of Human Ophthalmologists in Identifying Multiple Retinal Diseases in the Reader Study **eTable 10.** Κ Scores Between Retinal Artificial Intelligence Diagnosis System and Different Groups of Human Experts in the Reader Study **eTable 11.** Intergroup Correlation Between Ophthalmologists **eTable 12.** Efficiency Analysis of the DL Algorithm vs Human Ophthalmologists in Identifying Multiple Retinal Diseases in the Reader Study **eTable 13.** Performance of the Combination of Certified Ophthalmologists and the Retinal Artificial Intelligence Diagnosis System in the Reader Study **eTable 14.** Performance of the Combination of Junior Retinal Specialists and the Retinal Artificial Intelligence Diagnosis System in the Reader Study **eTable 15.** Performance of the Combination of Senior Retinal Specialists and the Retinal Artificial Intelligence Diagnosis System in the Reader Study
