# Supplementary material for: Artificial Intelligence for Screening of Multiple Retinal and Optic Nerve Diseases
Source: JAMA Netw Open. 2022 May 3;5(5):e229960. doi: 10.1001/jamanetworkopen.2022.9960 (PMC9066285; doi:10.1001/jamanetworkopen.2022.9960)
Supplement: Supplement. — eMethods. eFigure 1. The Overall Structure of Study eFigure 2. Distribution of Images in Prospective Validation Dataset eFigure 3. Performance of the RAIDS in Prospective Validation Dataset eFigure 4. Heatmap Visualization of Referral Diabetic Retinopathy eFigure 5. Heatmap Visualization of Referral Age-Related Macular Degeneration eFigure 6. Heatmap Visualization of Referral Possible Glaucoma eFigure 7. Heatmap Visualization of Pathological Myopia eFigure 8. Heatmap Visualization of Retinal Vein Occlusion eFigure 9. Heatmap Visualization of Macula Hole eFigure 10. Heatmap Visualization of Epiretinal Macular Membrane eFigure 11. Heatmap Visualization of Hypertensive Retinopathy eFigure 12. Heatmap Visualization of Myelinated Fibers eFigure 13. Heatmap Visualization of Retinitis Pigmentosa eFigure 14. Performance Comparison Between RAIDS and Human Ophthalmologists in Identifying Multiple Retinal Diseases eFigure 15. Performance of RAIDS-Human Ophthalmologist Combination in Identifying Multiple Retinal Diseases eTable 1. Development Dataset Distributions of Retinal Diseases and Camera Manufacturers eTable 2. Performance of the Retinal Artificial Intelligence Diagnosis System in the Internal Validation Dataset eTable 3. Performance of the Retinal Artificial Intelligence Diagnosis System (RAIDS) in Prospective Validation Dataset eTable 4. Performance of the Retinal Artificial Intelligence Diagnosis System in Identifying Multiple Retinal Diseases in East China eTable 5. Performance of the Retinal Artificial Intelligence Diagnosis System in Identifying Multiple Retinal Diseases in North China eTable 6. Performance of the Retinal Artificial Intelligence Diagnosis System Identifying Multiple Retinal Diseases in South China eTable 7. Performance of the Retinal Artificial Intelligence Diagnosis System in Identifying Multiple Retinal Diseases in West China eTable 8. Baseline Characteristics of Participants in the Reader Study eTable 9. Performance of Human Ophthalmologists in [file jamanetwopen-e229960-s001.pdf]

Supplemental Online Content

Dong L, He W, Zhang R, et al. Artificial intelligence for screening of multiple retinal and optic nerve diseases. *JAMA Netw Open*. 2022;5(2):e229960.
doi:10.1001/jamanetworkopen.2022.9960

eMethods.
eFigure 1. The Overall Structure of Study
eFigure 2. Distribution of Images in Prospective Validation Dataset
eFigure 3. Performance of the RAIDS in Prospective Validation Dataset
eFigure 4. Heatmap Visualization of Referral Diabetic Retinopathy
eFigure 5. Heatmap Visualization of Referral Age-Related Macular Degeneration
eFigure 6. Heatmap Visualization of Referral Possible Glaucoma
eFigure 7. Heatmap Visualization of Pathological Myopia
eFigure 8. Heatmap Visualization of Retinal Vein Occlusion
eFigure 9. Heatmap Visualization of Macula Hole
eFigure 10. Heatmap Visualization of Epiretinal Macular Membrane
eFigure 11. Heatmap Visualization of Hypertensive Retinopathy
eFigure 12. Heatmap Visualization of Myelinated Fibers
eFigure 13. Heatmap Visualization of Retinitis Pigmentosa
eFigure 14. Performance Comparison Between RAIDS and Human Ophthalmologists in Identifying Multiple Retinal Diseases
eFigure 15. Performance of RAIDS-Human Ophthalmologist Combination in Identifying Multiple Retinal Diseases
eTable 1. Development Dataset Distributions of Retinal Diseases and Camera Manufacturers
eTable 2. Performance of the Retinal Artificial Intelligence Diagnosis System in the Internal Validation Dataset
eTable 3. Performance of the Retinal Artificial Intelligence Diagnosis System (RAIDS) in Prospective Validation Dataset
eTable 4. Performance of the Retinal Artificial Intelligence Diagnosis System in Identifying Multiple Retinal Diseases in East China
eTable 5. Performance of the Retinal Artificial Intelligence Diagnosis System in Identifying Multiple Retinal Diseases in North China
eTable 6. Performance of the Retinal Artificial Intelligence Diagnosis System Identifying Multiple Retinal Diseases in South China
eTable 7. Performance of the Retinal Artificial Intelligence Diagnosis System in Identifying Multiple Retinal Diseases in West China
eTable 8. Baseline Characteristics of Participants in the Reader Study
eTable 9. Performance of Human Ophthalmologists in Identifying Multiple Retinal Diseases in the Reader Study
eTable 10. K Scores Between Retinal Artificial Intelligence Diagnosis System and Different Groups of Human Experts in the Reader Study
eTable 11. Intergroup Correlation Between Ophthalmologists
eTable 12. Efficiency Analysis of the DL Algorithm vs Human Ophthalmologists in Identifying Multiple Retinal Diseases in the Reader Study
eTable 13. Performance of the Combination of Certified Ophthalmologists and the Retinal Artificial Intelligence Diagnosis System in the Reader Study
eTable 14. Performance of the Combination of Junior Retinal Specialists and the Retinal Artificial Intelligence Diagnosis System in the Reader Study
eTable 15. Performance of the Combination of Senior Retinal Specialists and the Retinal Artificial Intelligence Diagnosis System in the Reader Study

This supplemental material has been provided by the authors to give readers additional information about their work.

eMethods.

(a) Evaluation metric calculation

From the point of classification methods, RAIDS was essentially a multi-label classification system. Therefore, the evaluation of RAIDS followed the commonly-used evaluation method, which took the evaluation of each abnormality as binary-classification evaluation. The binary evaluation was derived from the concept of confusion matrix. For each kind of abnormality, if the abnormality was present in the ground truth of a sample image, then this image would be positive related to this abnormality, otherwise it was negative. So did the prediction. Having obtained the confusion matrix for each kind of abnormality, the metrics of accuracy, sensitivity and specificity were calculated following the formulas:

GT \ Pred	neg	pos
neg	TN	FP
pos	FN	TP

$$acc = \frac{TN + TP}{TN + TP + FN + FP}$$

$$sens = \frac{TP}{TP + FN}$$

$$spec = \frac{TN}{TN + FP}$$

To be noted here, area under curve (AUC) was not applicable to the evaluation of RAIDS. RAIDS was based a multi-task multi-label classification model. For each sub-task, it was feasible to calculate AUC for each class or abnormality related to this sub-task. However, as some kinds of abnormalities were determined by two of the sub-tasks, e.g. macula hole was determined by both macula-branch and general-branch, which resulted in two possibilities for macula hole, it was theoretically infeasible to draw a two-dimensional ROC curve for the whole multi-task model, and neither do the AUC which was derived from ROC curve as the area under the curve.

© 2022 Dong L et al. *JAMA Network Open.*

(b) Diagnosis criteria of fundus images in health care screening

1. Normal fundus: Rosy optic disc, with the cup disc ratio of 0.2-0.6; the arteriovenous ratio between 2:3 and 1:2; no sign of bleeding and other obvious lesions; tessellation fundus; small drusen < 125 μm in greatest linear diameter.

2. Referable diabetic retinopathy: defined as a diabetic retinopathy severity level of moderate non-proliferative diabetic retinopathy or worse, diabetic macular edema.[1]

3. Referable AMD: defined as the presence of intermediate AMD (numerous medium-sized drusen, 1 large drusen ≥125 μm in greatest linear diameter; geographical atrophy; advanced AMD. [2]

4. Referable possible glaucoma: defined as a ratio of vertical cup to disc diameter ≥0.7, focal thinning or notching of the neuroretinal rim, optic disc hemorrhages, or localized retinal nerve fiber layer defects.[3]

5. Pathological myopia: defined as diffuse chorioretinal atrophy or greater degree of atrophy.[4]

6. Retinal vein occlusion: defined as intraretinal hemorrhages and dilated tortuous retinal vasculature. Both central retinal vein occlusion and branch retinal vein occlusion were labelled in this classification.[5]

7. Hypertensive retinopathy: Obvious arterial narrowing with focal irregularities and/or retinal hemorrhages and/or exudates and/or optic nerve head swelling

8. Retinitis pigmentosa: defined as mid-peripheral ‘bone spicule’ retinal pigmentation, waxy pallor of the optic disc, arteriolar attenuation. [6]

9. **Macular hole**: defined as yellow spot or a yellow circle in the fovea. (Gass classification stage 2 or above)

10. Macular Epiretinal Membranes: defined as glistening light reflex, retinal striae or/and tortuous, straightened, obscured vessels.[7]

© 2022 Dong L et al. *JAMA Network Open.*

11. Myelinated retinal nerve fiber layer: defined as distinct peripapillary white striated patch with feathered borders approximately one disc diameter in size or larger.

Reference
[1] Grading Diabetic Retinopathy from Stereoscopic Color Fundus Photographs—An Extension of the Modified Airlie House Classification:eTDRS Report Number 10 [J]. Ophthalmology, 1991, 98(5, Supplement): 786-806.
[2] Ferris F L, III, Wilkinson C P, Bird A,eT al. Clinical Classification of Age-related Macular Degeneration [J]. Ophthalmology, 2013, 120(4): 844-851.
[3] Ting D S W, Cheung C Y-L, Lim G,eT al. Development and Validation of a Deep Learning System for Diabetic Retinopathy and Related Eye Diseases Using Retinal Images From Multiethnic Populations With Diabetes [J]. JAMA, 2017, 318(22): 2211-2223.
[4] Ohno-Matsui K, Kawasaki R, Jonas J B,eT al. International Photographic Classification and Grading System for Myopic Maculopathy [J]. Am J Ophthalmol, 2015, 159(5): 877-883.e877.
[5] Wong T Y, Scott I U. Retinal-Vein Occlusion [J]. New England Journal of Medicine, 2010, 363(22): 2135-2144.
[6] Hartong D T, Berson E L, Dryja T P. Retinitis pigmentosa [J]. The Lancet, 2006, 368(9549): 1795-1809.
[7] Bu S-C, Kuijer R, Li X-R,eT al. IDIOPATHIC EPIRETINAL MEMBRANE [J]. Retina, 2014, 34(12):
[8] Biousse V, Newman N J, Najjar R P,eT al. Optic Disc Classification by Deep Learning versus Expert Neuro-Ophthalmologists [J]. Annals of Neurology, 2020, 88(4): 785-795.

(c) List of the location of health screening centers and the fundus cameras used in each center.

Subregions	Province	Camera Model	iKang Corporation public health screening centers (City)
East China	Shanghai	TRC-NW200	Shanghai Yuanhua Medical Centre of iKang Guobin Healthcare Group (Shanghai, China)
East China	Shanghai	CR-2 AF	Shanghai Jun'an Medical Centre of iKang Healthcare Group (Shanghai, China)
East China	Shanghai	CR-2 AF	Shanghai Wangzu Medical Centre of iKang Guobin Healthcare Group (Shanghai, China)
East China	Shanghai	CR-2 AF	Shanghai Zhiwei Medical Centre of iKang Guobin Healthcare Group (Shanghai, China)
East China	Shanghai	CR-2 AF	Shanghai Yipin Medical Centre of iKang Guobin Healthcare Group (Shanghai, China)
East China	Shanghai	CR-2 AF	Shanghai Fukang Medical Centre of iKang Guobin Healthcare Group (Shanghai, China)
East China	Shanghai	CR-2 AF	Shanghai Binming Medical Centre of iKang Guobin Healthcare Group (Shanghai, China)
East China	Shanghai	CR-2 AF	Shanghai Waizhitan Medical Centre of iKang Guobin Healthcare Group (Shanghai, China)
East China	Shanghai	CR-2 AF	Shanghai Renren Medical Centre of iKang Guobin Healthcare Group (Shanghai, China)

© 2022 Dong L et al. *JAMA Network Open.*

East China	Anhui	CR-2 AF	Hefei Medical Centre of iKang Healthcare Group (Hefei, China)
East China	Anhui	CR-2 AF	Wuhu Medical Centre of iKang Guobin Healthcare Group (Wuhu, China)
East China	Jiangsu	CR-2 AF	Nanjing Junan Medical Centre of iKang Guobin Healthcare Group (Nanjing, China)
East China	Jiangsu	CR-2 AF	Nanjing Xinjie Medical Centre of iKang Guobin Healthcare Group (Nanjing, China)
East China	Jiangsu	CR-2 AF	Wuxi Medical Centre of iKang Healthcare Group (Wuxi, China)
East China	Jiangsu	CR-2 AF	Hangzhou Wenhui Medical Centre of iKang Healthcare Group (Hangzhou, China)
East China	Jiangsu	CR-2 AF	Hangzhou Zhuoyue Medical Centre of iKang Healthcare Group (Hangzhou, China)
East China	Jiangsu	CR-2 AF	Hangzhou Junan Medical Centre of iKang Guobin Healthcare Group (Hangzhou, China)
East China	Jiangsu	CR-2 AF	Suzhou Medical Centre of iKang Guobin Healthcare Group (Suzhou, China)
East China	Jiangsu	CR-2 AF	Suzhou Zhuoyue Medical Centre of iKang Healthcare Group (Suzhou, China)
East China	Jiangsu	CR-2 AF	Zhenjiang Wenguang Medical Centre of iKang Healthcare Group (Zhenjiang, China)
East China	Jiangsu	CR-2 AF	Jiangyin LingangMedical Centre of iKang Healthcare Group (Jiangyin, China)
East China	Zhejiang	CR-2 AF	Ningbo Haishu Medical Centre of iKang Guobin Healthcare Group (Ningbo, China)
East China	Hubei	CR-2 AF	Yichang Zhuoyue Medical Centre of iKang Healthcare Group (Yichang, China)
East China	Hubei	CR-2 AF	Wuhan Jindun Medical Centre of iKang Guobin Healthcare Group (Wuhan, China)
East China	Hubei	CR-2 AF	Wuhan Zhuoyue Medical Centre of iKang Healthcare Group (Wuhan, China)
**Subregions**	**Province**	**Camera Model**	**iKang Corporation public health screening centers (City)**
North China	Jilin	CR-2 AF	Changchun Jianshe Medical Centre of iKang Guobin Healthcare Group (Changchun, China)
North China	Tianjin	CR-2 AF	Tianjin Fenghui Medical Centre of iKang Guobin Healthcare Group (Tianjin, China)
North China	Tianjin	CR-2 AF	Tianjin Dongrun Medical Centre of iKang Guobin Healthcare Group (Tianjin, China)
North China	Tianjin	CR-2 AF	Tianjin Heping Medical Centre of iKang Healthcare Group (Tianjin, China)
North China	Tianjin	CR-2 AF	Tianjin Hexi Medical Centre of iKang Guobin Healthcare Group (Tianjin, China)
North China	Tianjin	CR-2 AF	Tianjin Yuecheng Medical Centre of iKang Guobin Healthcare Group (Tianjin, China)
North China	Shandong	CR-2 AF	Weihai Ciming Medical Centre of iKang Guobin Healthcare Group (Weihai, China)
North China	Shandong	CR-2 AF	Jinan Zhuoyue Medical Centre of iKang Healthcare Group (Jinan, China)
North China	Shandong	CR-2 AF	Weifang Ciming Medical Centre of iKang Guobin Healthcare Group (Weifang, China)
North China	Shandong	CR-2 AF	Yantai Medical Centre of iKang Healthcare Group (Yantai, China)
North China	Shandong	CR-2 AF	Qingdao Zhuoyue Medical Centre of iKang Healthcare Group (Qingdao, China)
North China	Liaoning	TRC-NW300	Shenyang Jinai Medical Centre of iKang Guobin Healthcare Group (Shenyang, China)

© 2022 Dong L et al. *JAMA Network Open.*

North China	Liaoning	CR-2 AF	Shenyang Shenhe Medical Centre of iKang Guobin Healthcare Group (Shenyang, China)
North China	Beijing	CR-2 AF	Beijing Tongren hospital (Beijing, China)
North China	Beijing	CR-2 AF	Beijing Friendship Hospital (Beijing, China)
North China	Shanxi	Nonmyd α-DⅢ	Xi'an Qujiang Medical Centre of iKang Guobin Healthcare Group (Xi'an, China)
North China	Shanxi	Nonmyd α-DⅢ	Xi'an Weiyang Medical Centre of iKang Guobin Healthcare Group (Xi'an, China)
North China	Shanxi	Nonmyd α-DⅢ	Xi'an Lianhu Medical Centre of iKang Healthcare Group (Xi'an, China)
South China	Guangdong	CR-2 AF	Dongguan Songshan Medical Centre of iKang Healthcare Group (Dongguan, China)
South China	Guangdong	CR-2 AF	Dongguan Caifu Medical Centre of iKang Guobin Healthcare Group (Dongguan, China)
South China	Guangdong	CR-2 AF	Foshan Medical Centre of iKang Healthcare Group (Foshan, China)
South China	Guangdong	CR-2 AF	Guangzhou Medical Centre of iKang Guobin Healthcare Group (Guangzhou, China)
South China	Guangdong	CR-2 AF	Guangzhou Junan Medical Centre of iKang Healthcare Group (Guangzhou, China)
South China	Guangdong	CR-2 AF	Guangzhou Nantian Medical Centre of iKang Healthcare Group (Guangzhou, China)
South China	Guangdong	CR-2 AF	Guangzhou Tianhe Medical Centre of iKang Healthcare Group (Guangzhou, China)
South China	Shenzhen	NT-2000	Shenzhen Zhuoyue Medical Centre of iKang Healthcare Group (Shenzhen, China)
South China	Shenzhen	CR-2 AF	Shenzhen Xinglin Medical Centre of iKang Guobin Healthcare Group (Shenzhen, China)
South China	Hunan	CR-2 AF	Changsha shuangta Medical Centre of iKang Healthcare Group (Changsha, China)
**Subregions**	**Province**	**Camera Model**	**iKang Corporation public health screening centers (City)**
South China	Fujian	CR-2 AF	Fuzhou Gulou Medical Centre of iKang Guobin Healthcare Group (Fuzhou, China)
West China	Chongqing	CR-2 AF	Chongqing Zhuoyue Medical Centre of iKang Healthcare Group (Chongqing, China)
West China	Sichuan	CR-2 AF	Chengdu Anshengmei Medical Centre of iKang Guobin Healthcare Group (Chengdu, China)
West China	Sichuan	CR-2 AF	Chengdu Luomashi Medical Centre of iKang Guobin Healthcare Group (Chengdu, China)
West China	Sichuan	CR-2 AF	Chengdu Hongzhaobi Medical Centre of iKang Guobin Healthcare Group (Chengdu, China)
West China	Sichuan	CR-2 AF	Mianyang Medical Centre of iKang Guobin Healthcare Group (Mianyang, China)
West China	Ningxia	CR-2 AF	Guyuan Medical Centre of iKang Guobin Healthcare Group (Guyuan, China)
West China	Ningxia	CR-2 AF	Yinchuan Medical Centre of iKang Guobin Healthcare Group (Yinchuan, China)
West China	Guizhou	CR-2 AF	Liupanshui Medical Centre of iKang Guobin Healthcare Group (Liupanshui, China)
West China	Guizhou	CR-2 AF	Kaili Medical Centre of iKang Guobin Healthcare Group (Kaili, China)
West China	Guizhou	CR-2 AF	Bijie Medical Centre of iKang Guobin Healthcare Group (Bijie, China)
West China	Guizhou	CR-2 AF	Guizhou Zhuoyue Medical Centre of iKang Guobin Healthcare Group (Guiyang, China)

© 2022 Dong L et al. *JAMA Network Open.*

(d) Detailed information of the 45-expert panel in prospective validation dataset

	Years of experience		
Reads per year	5-9	10-14	15-19
10000-30000	3	1	4
30000-50000	5	4	1
50000-70000	12	4	0
>70000	9	2	0

(e) Detailed information of the 6-expert panel in retrospective test dataset – Reader Study

Years of experience	
15-19	≥20
4	2

© 2022 Dong L et al. *JAMA Network Open.*

eFigure 1. The Overall Structure of Study

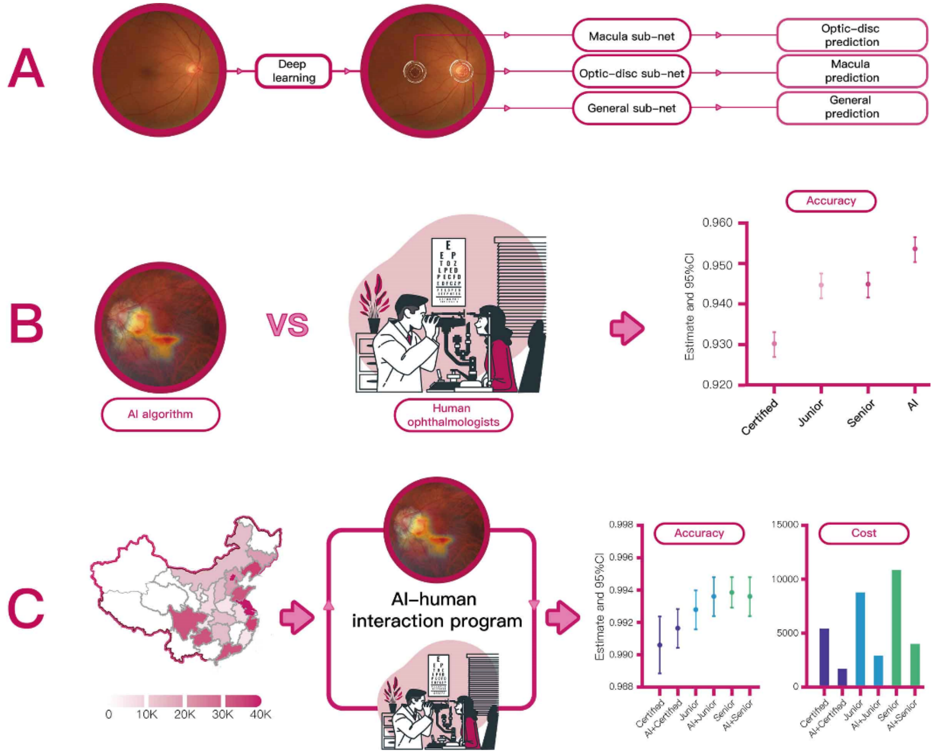

A. The structure of the proposed multi-task classification method; B. Schematic diagram of the prospective study; C. Schematic diagram of the reader study.

© 2022 Dong L et al. *JAMA Network Open.*

eFigure 2. Distribution of Images in Prospective Validation Dataset

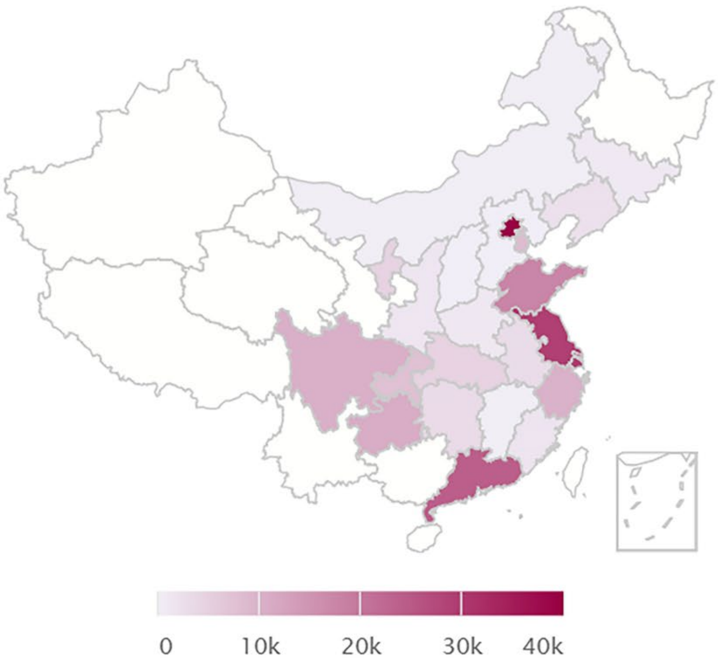

© 2022 Dong L et al. *JAMA Network Open.*

eFigure 3. Performance of the RAIDS in Prospective Validation Dataset

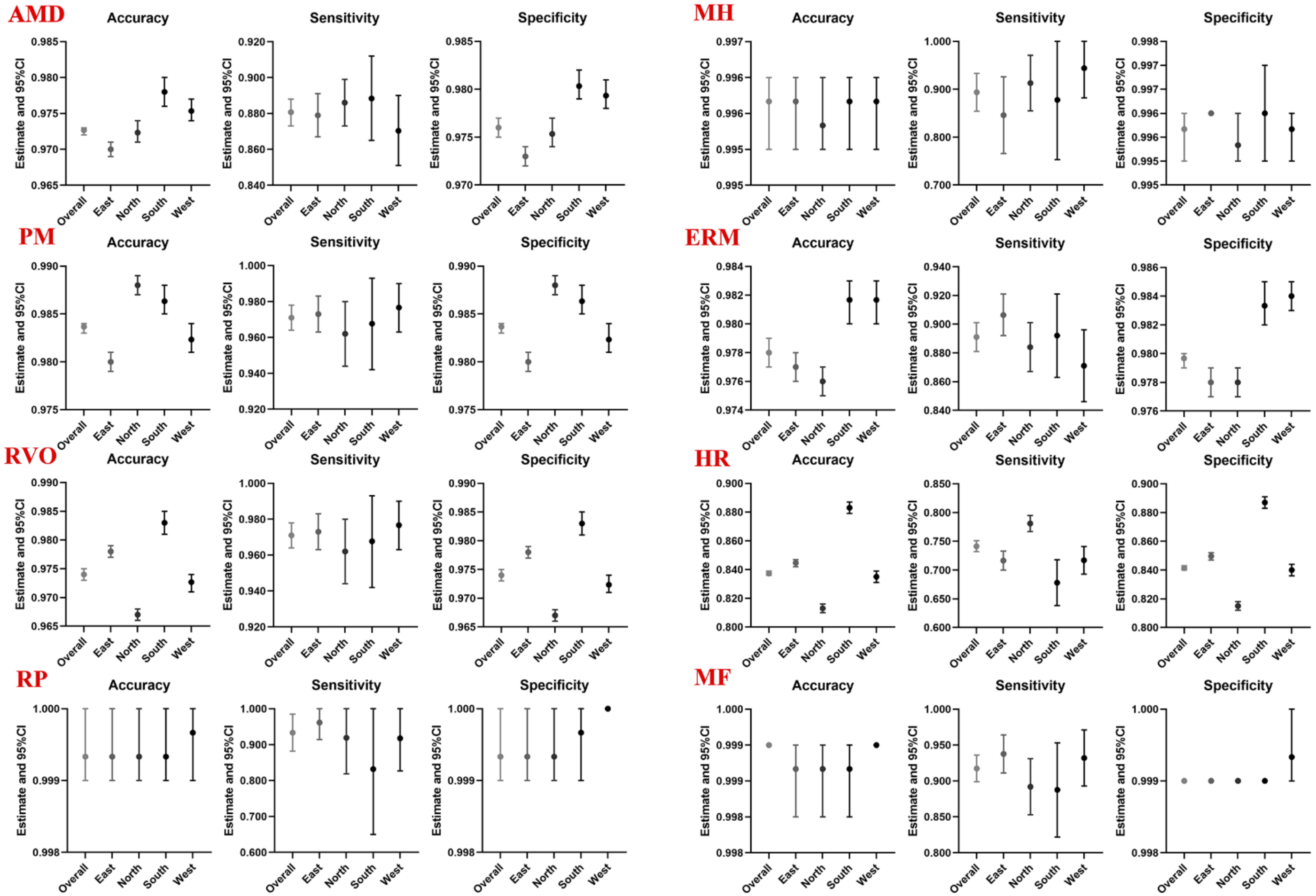

AMD, age-related macular degeneration; PM, pathologic myopia; ERM, epiretinal macular membrane; HR, hypertensive retinopathy; RVO, retinal vein occlusion; MH, macula hole; MF, myelinated fibers; RP, retinitis pigmentosa.

© 2022 Dong L et al. *JAMA Network Open.*

eFigure 4. Heatmap Visualization of Referral Diabetic Retinopathy

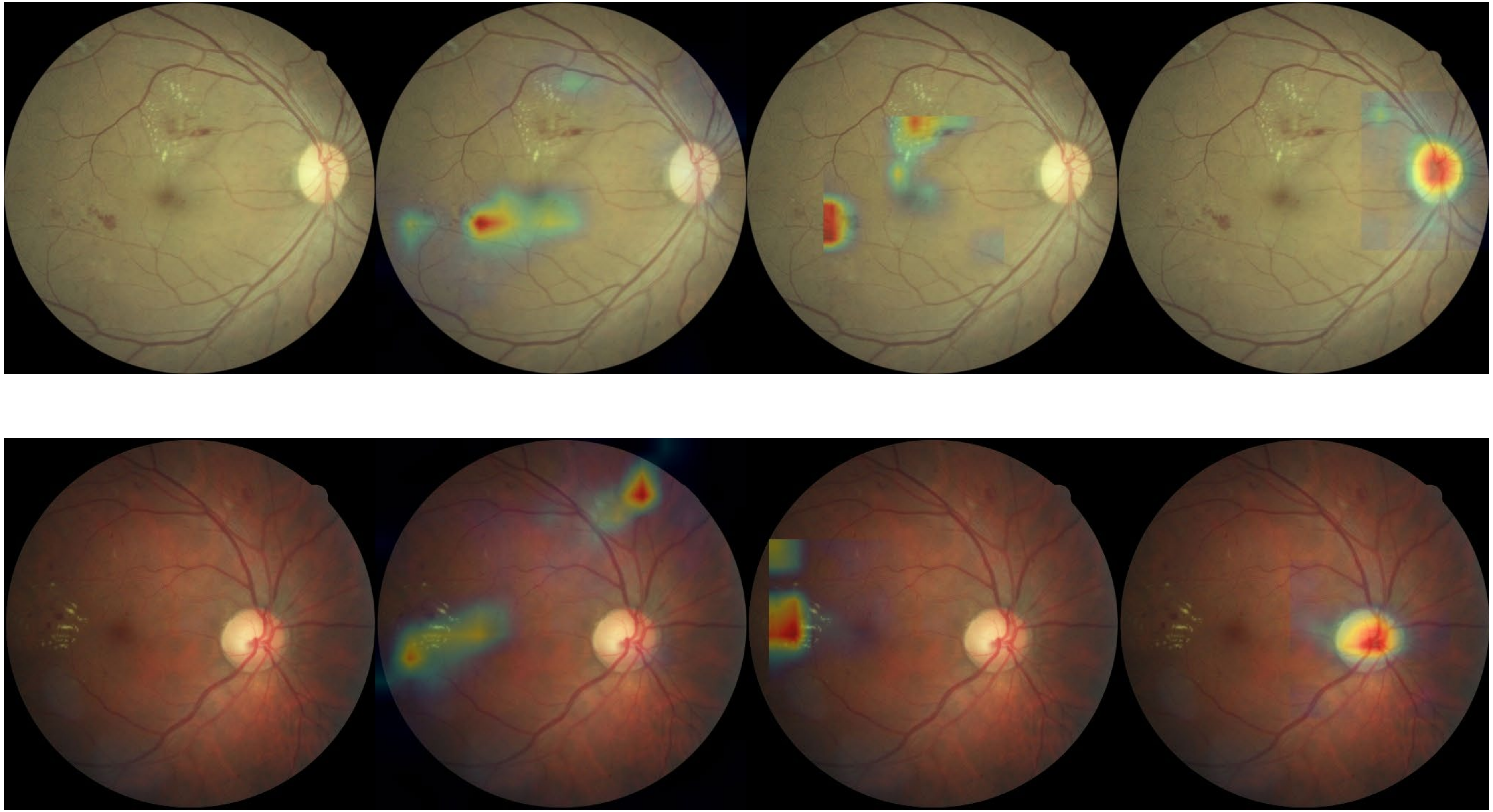

© 2022 Dong L et al. *JAMA Network Open.*

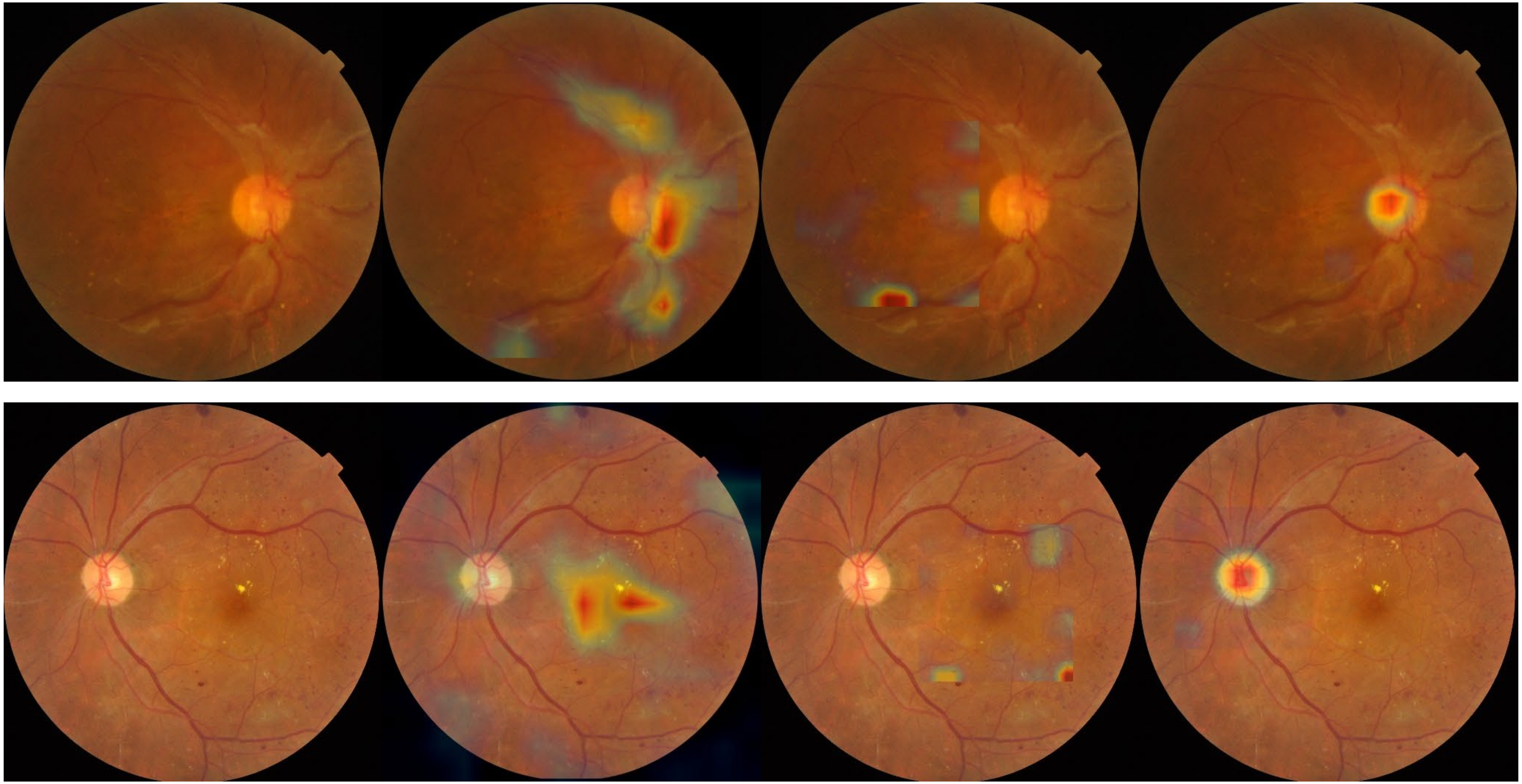

Heatmap demonstrating representative lesions, shown in original fundus image (first column), general heatmap (second column), macula heatmap (third column), and optic-disc heatmap (last column)

© 2022 Dong L et al. *JAMA Network Open.*

eFigure 5. Heatmap Visualization of Referral Age-Related Macular Degeneration

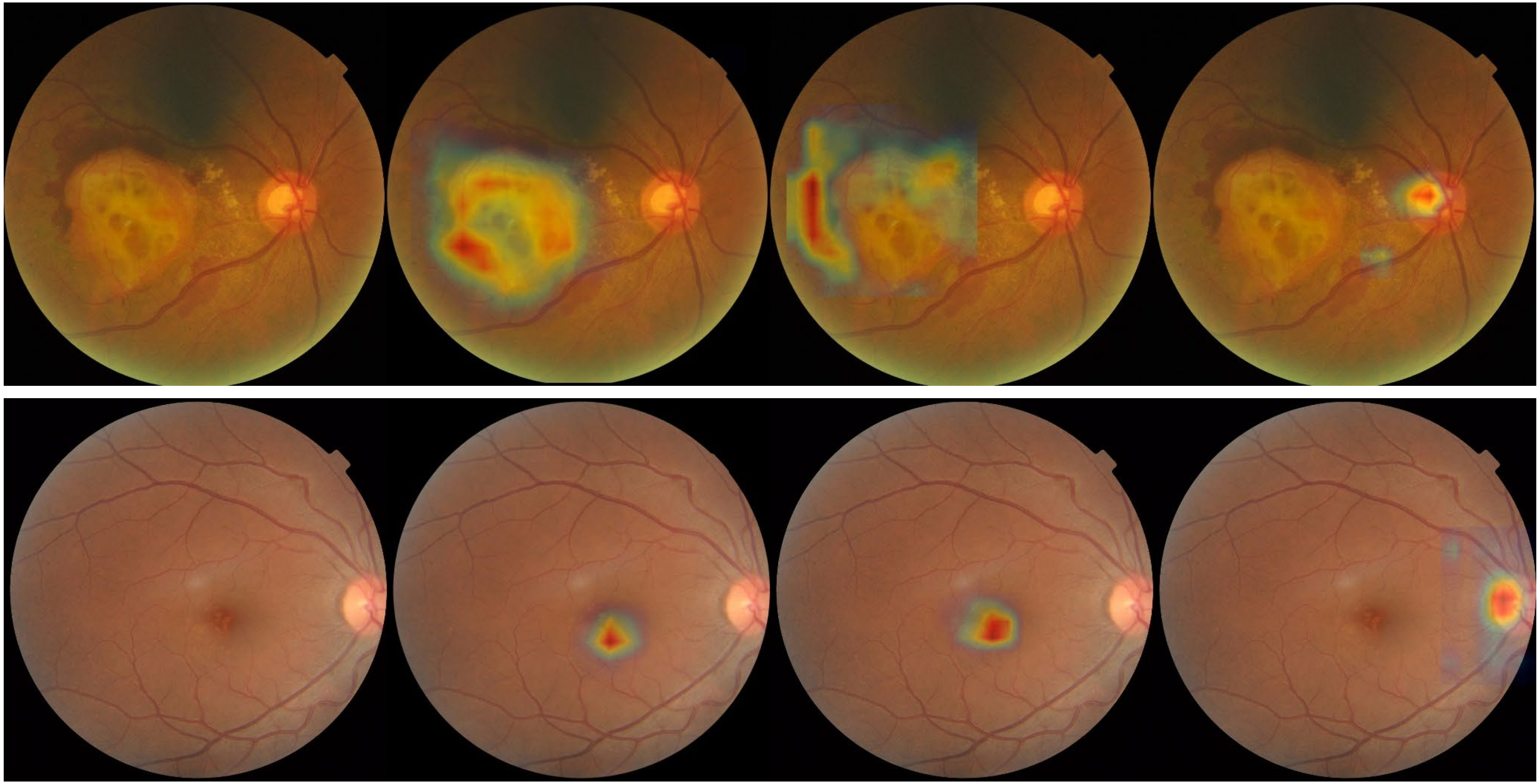

© 2022 Dong L et al. *JAMA Network Open.*

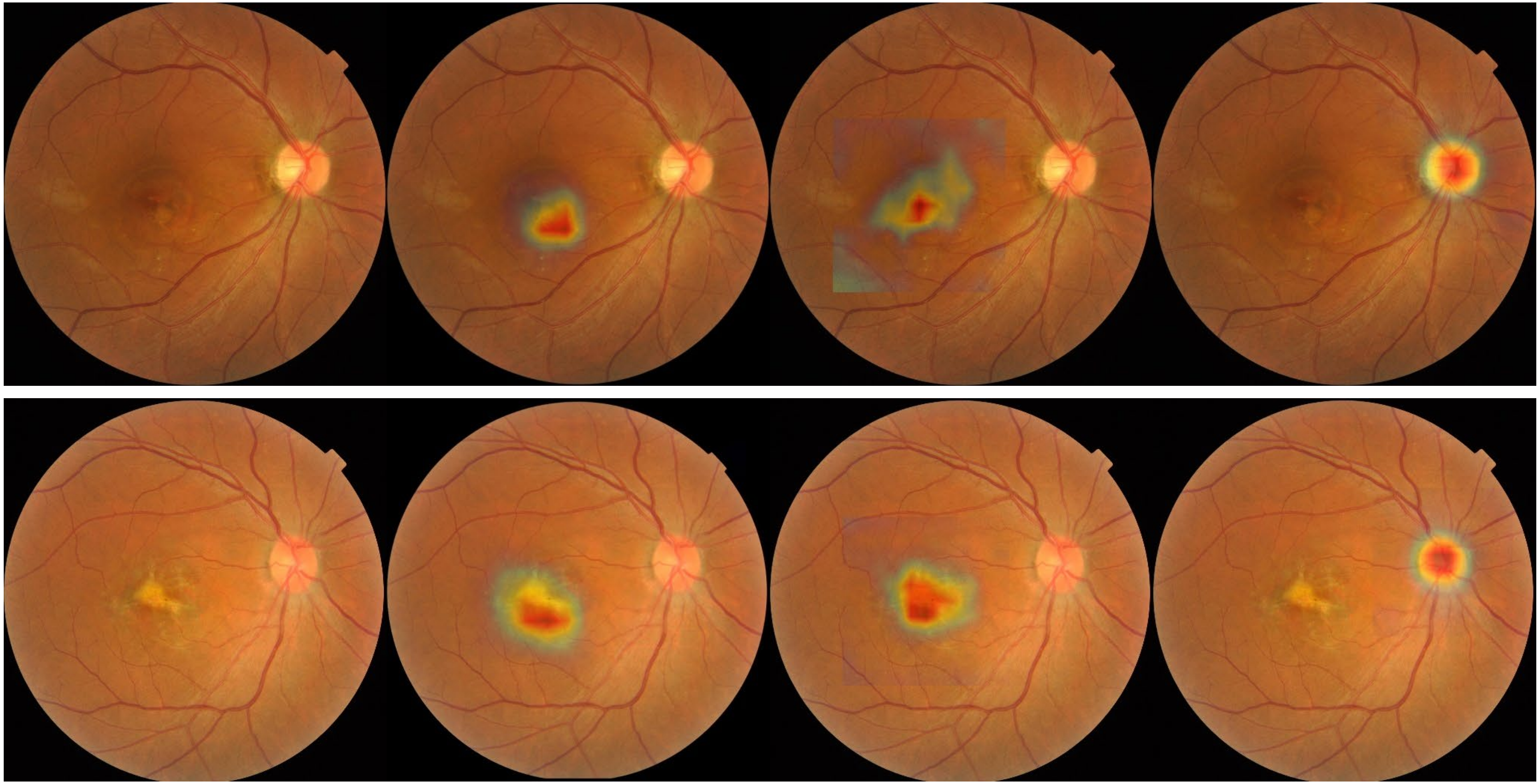

Heatmap demonstrating representative lesions, shown in original fundus image (first column), general heatmap (second column), macula heatmap (third column), and optic-disc heatmap (last column)

© 2022 Dong L et al. *JAMA Network Open.*

eFigure 6. Heatmap Visualization of Referral Possible Glaucoma

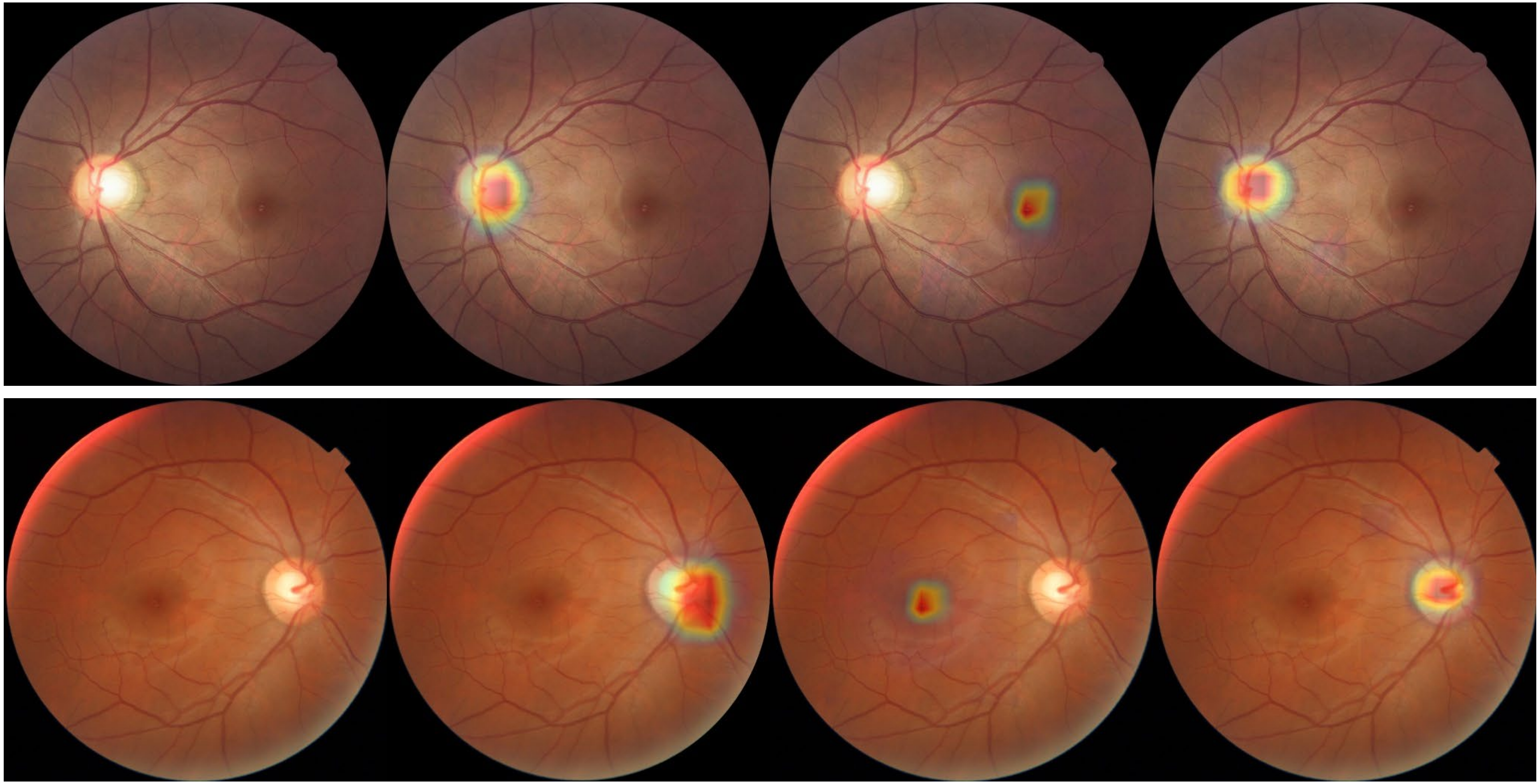

© 2022 Dong L et al. *JAMA Network Open.*

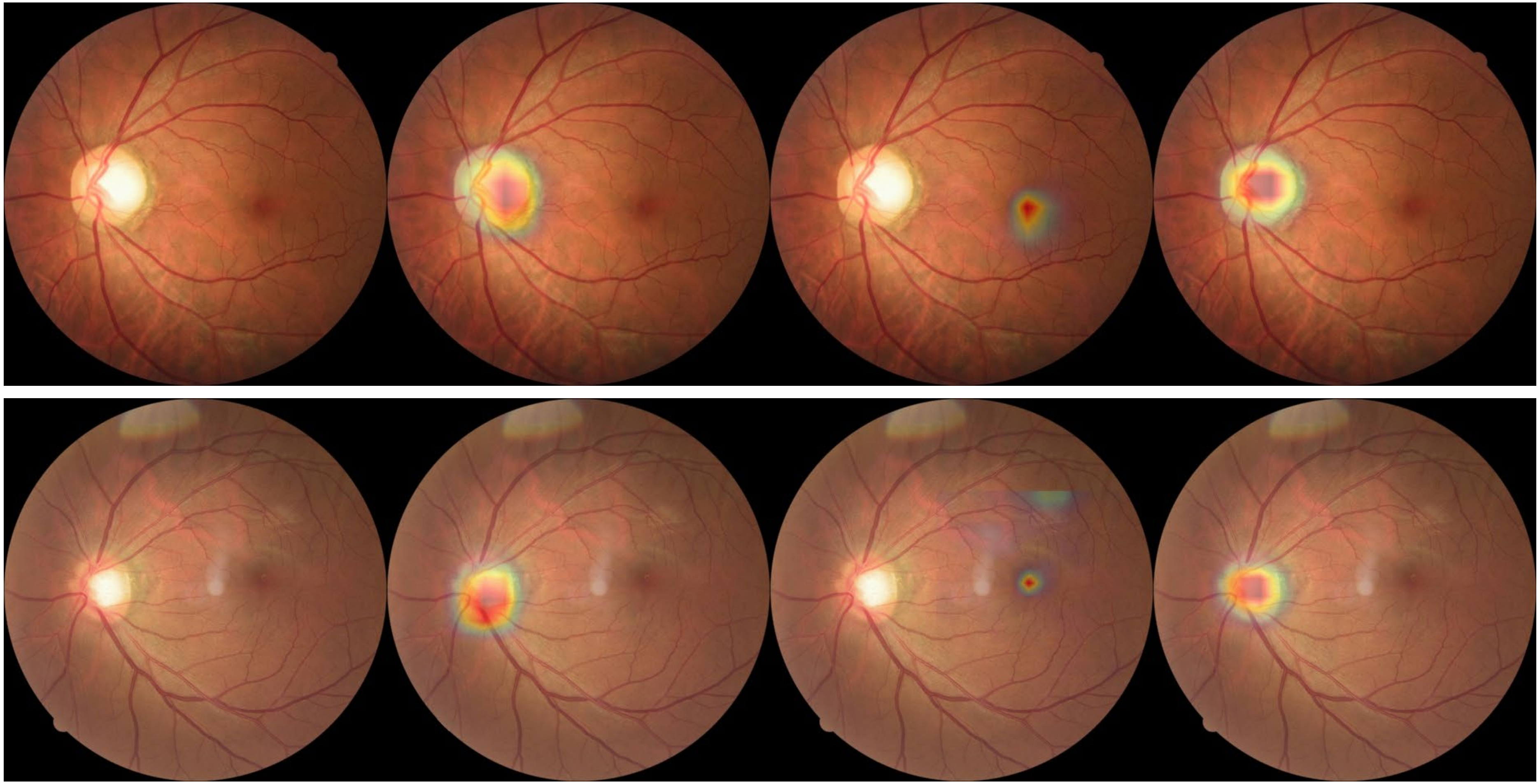

Heatmap demonstrating representative lesions, shown in original fundus image (first column), general heatmap (second column), macula heatmap (third column), and optic-disc heatmap (last column)

© 2022 Dong L et al. *JAMA Network Open.*

eFigure 7. Heatmap Visualization of Pathological Myopia

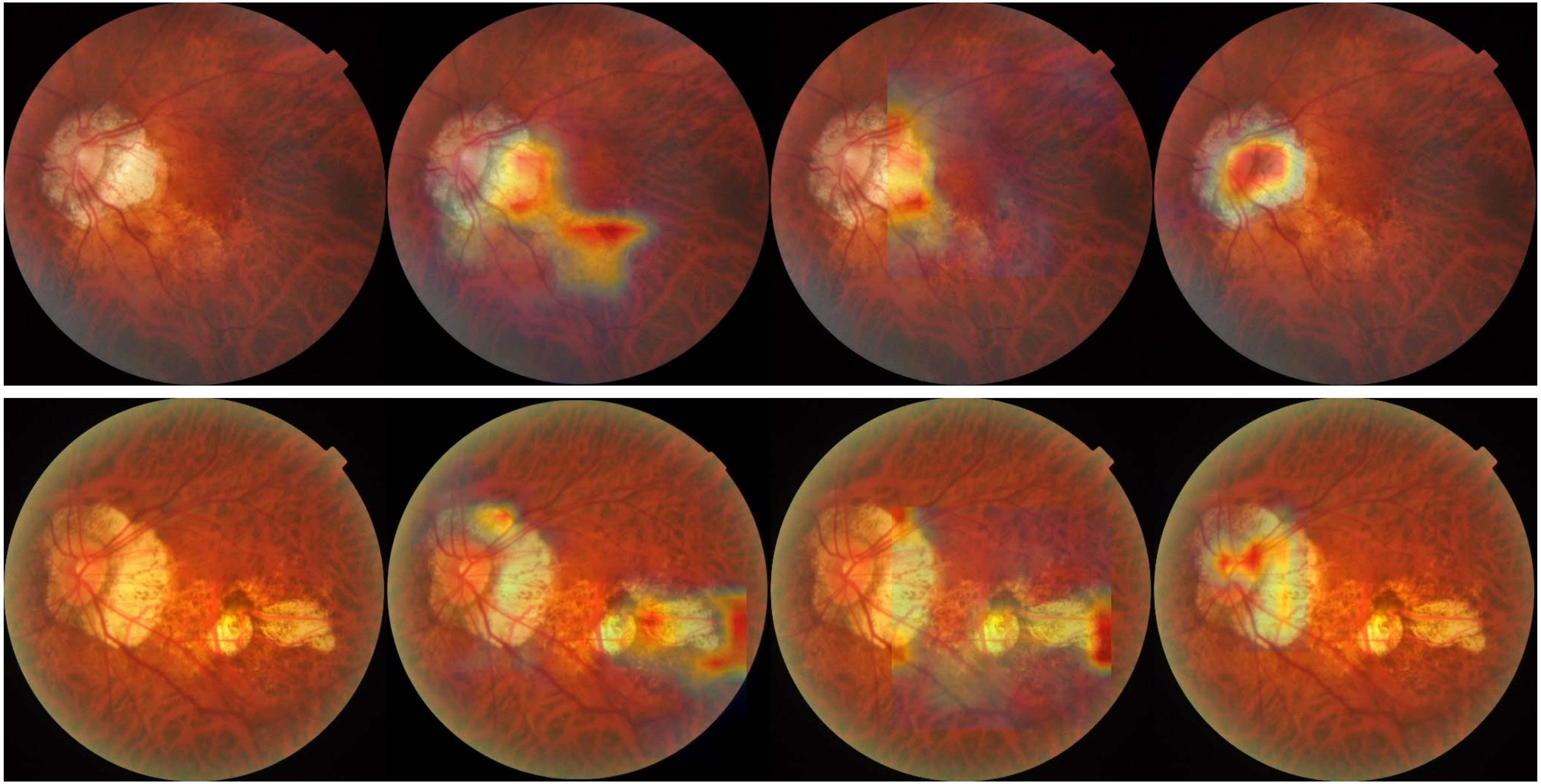

© 2022 Dong L et al. *JAMA Network Open.*

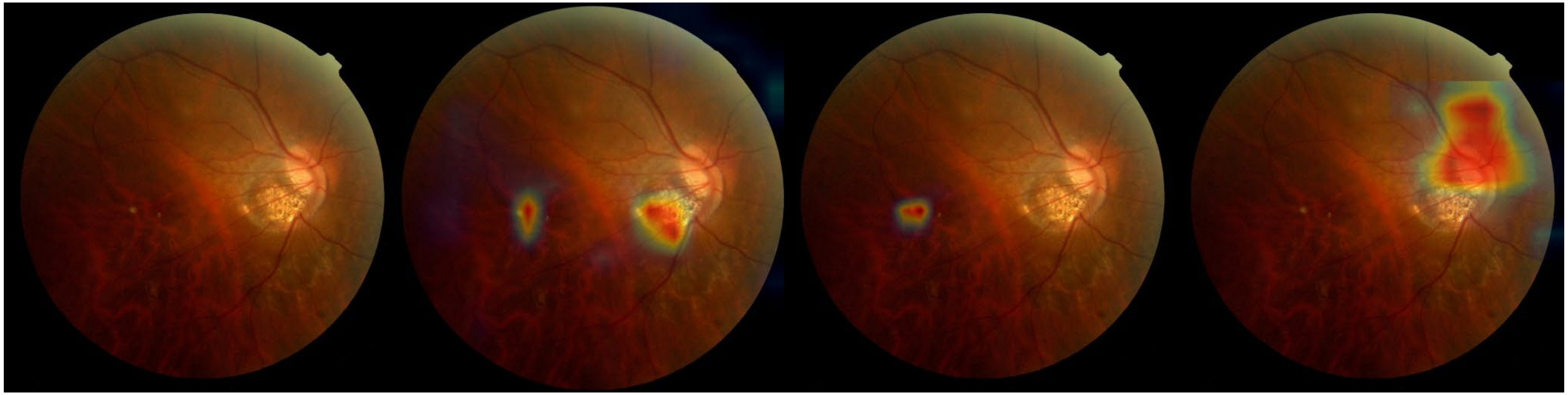

Heatmap demonstrating representative lesions, shown in original fundus image (first column), general heatmap (second column), macula heatmap (third column), and optic-disc heatmap (last column)

© 2022 Dong L et al. *JAMA Network Open.*

eFigure 8. Heatmap Visualization of Retinal Vein Occlusion

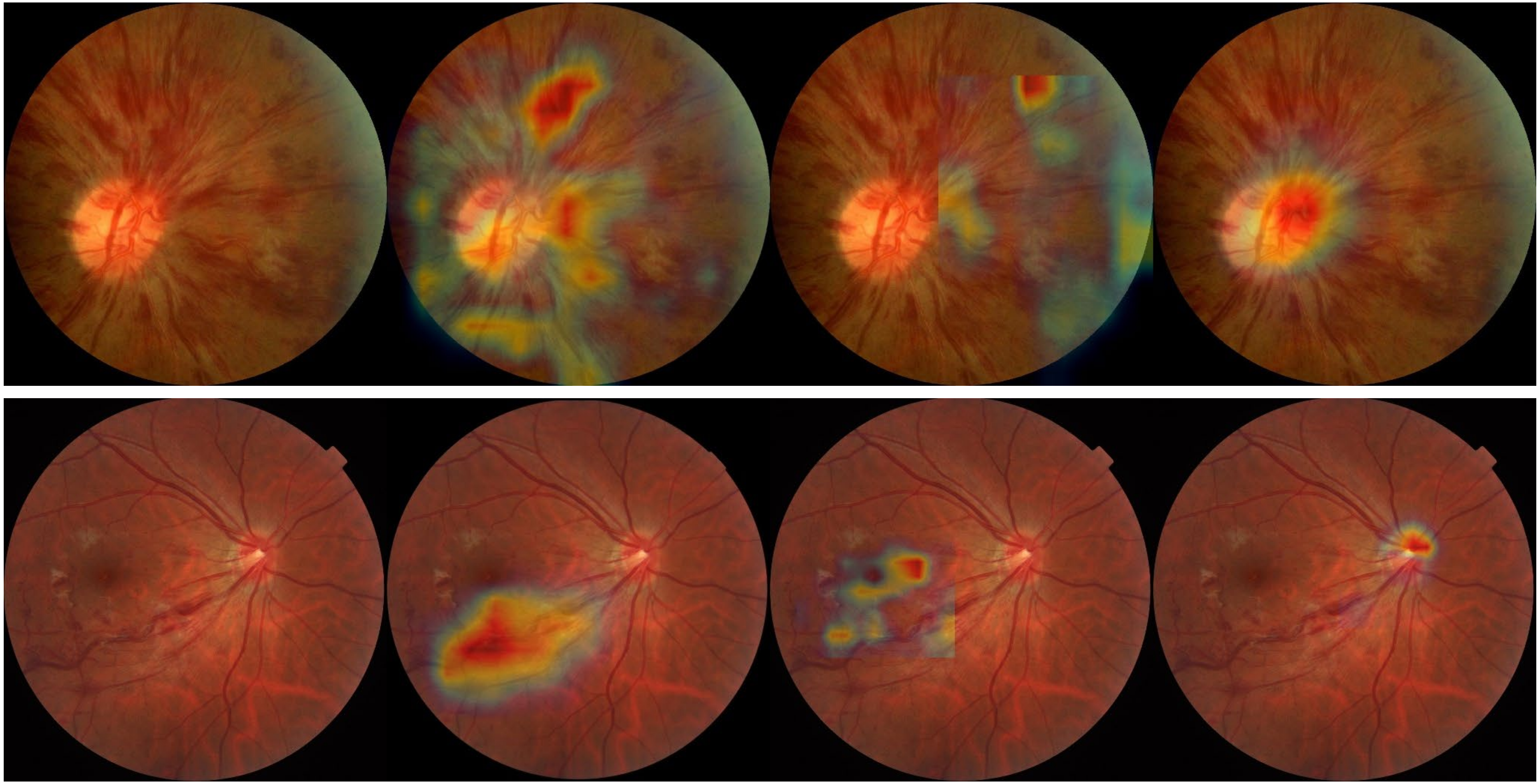

Heatmap demonstrating representative lesions, shown in original fundus image (first column), general heatmap (second column), macula heatmap (third column), and optic-disc heatmap (last column)

© 2022 Dong L et al. *JAMA Network Open.*

eFigure 9. Heatmap Visualization of Macula Hole

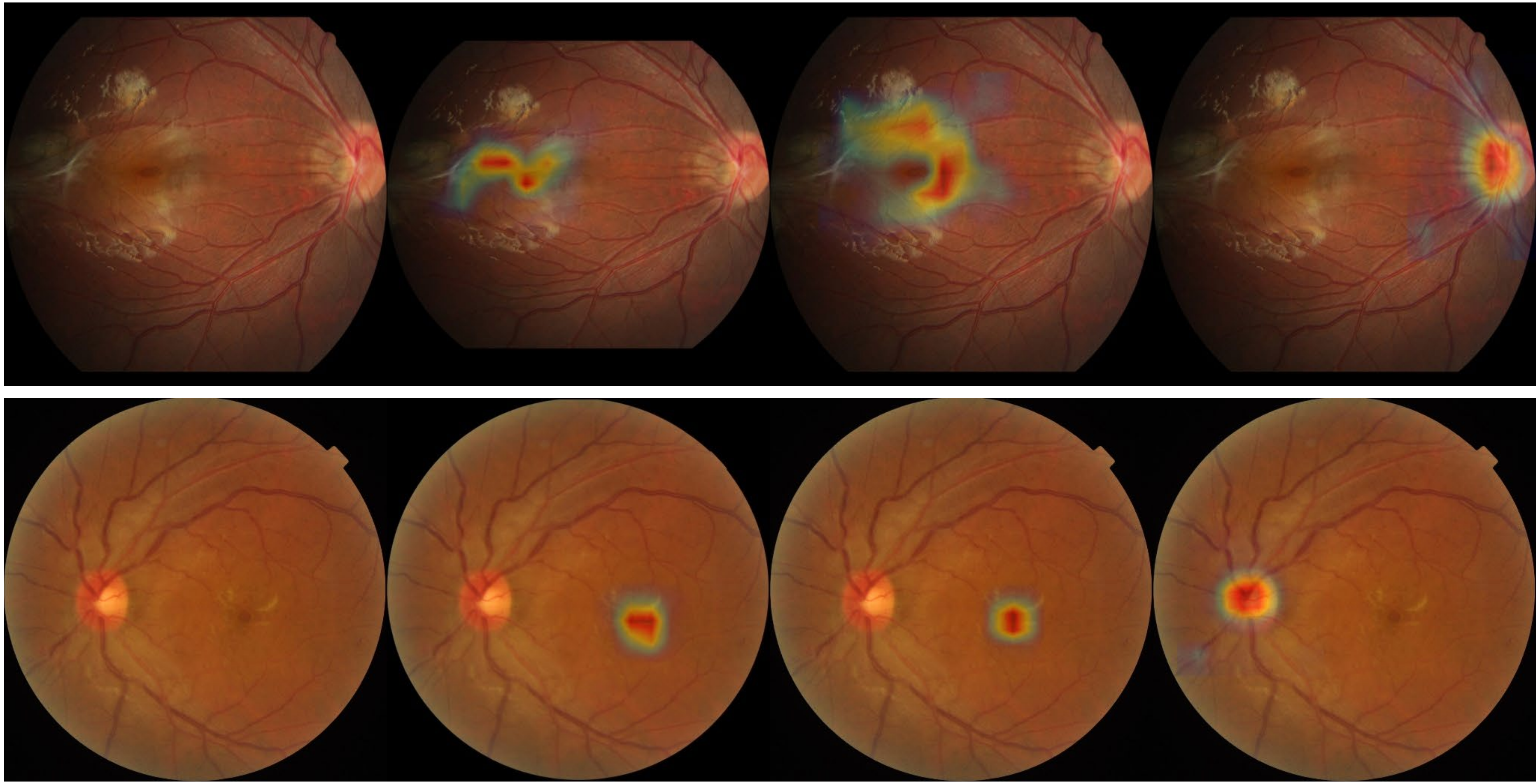

© 2022 Dong L et al. *JAMA Network Open.*

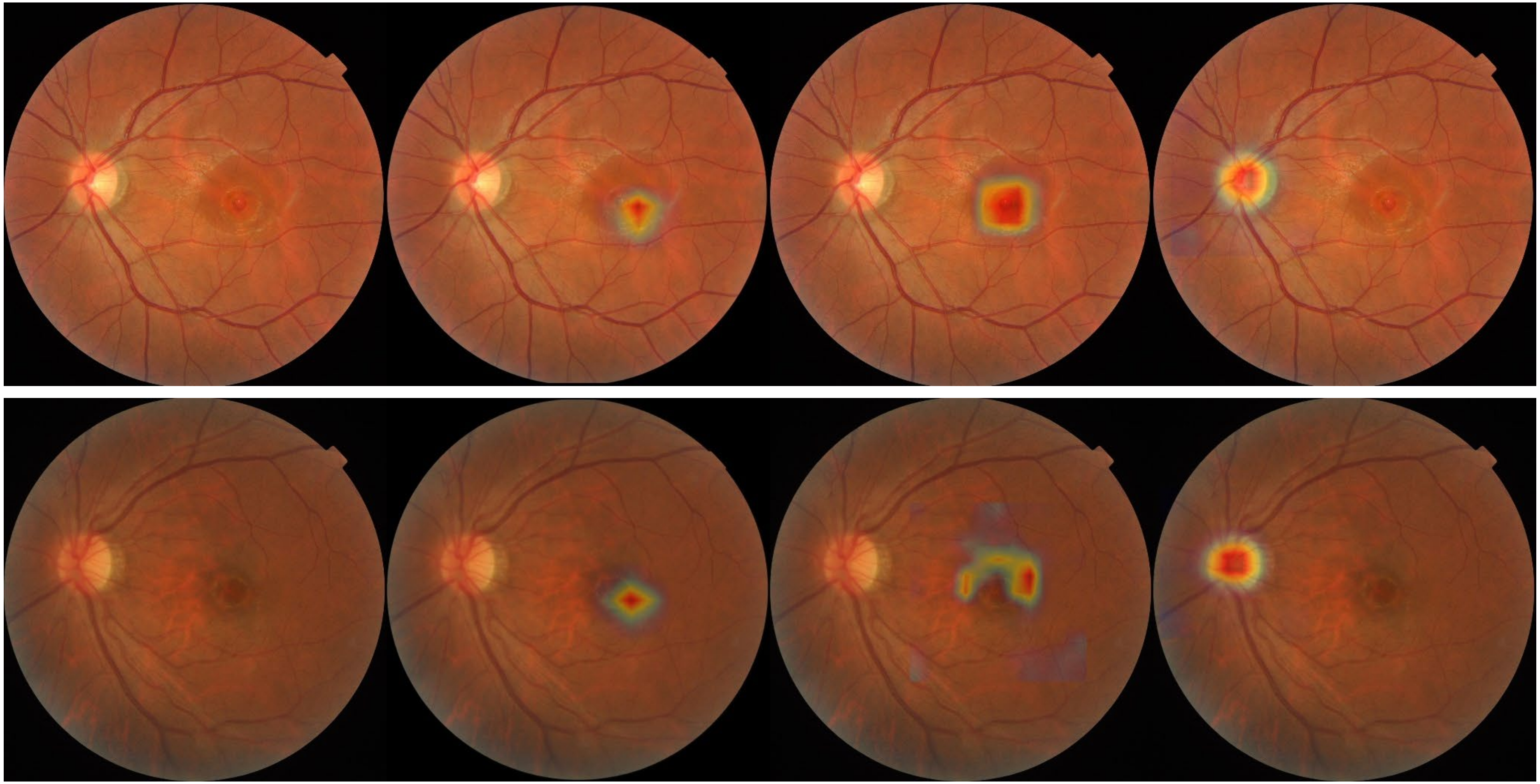

Heatmap demonstrating representative lesions, shown in original fundus image (first column), general heatmap (second column), macula heatmap (third column), and optic-disc heatmap (last column)

© 2022 Dong L et al. *JAMA Network Open.*

eFigure 10. Heatmap Visualization of Epiretinal Macular Membrane

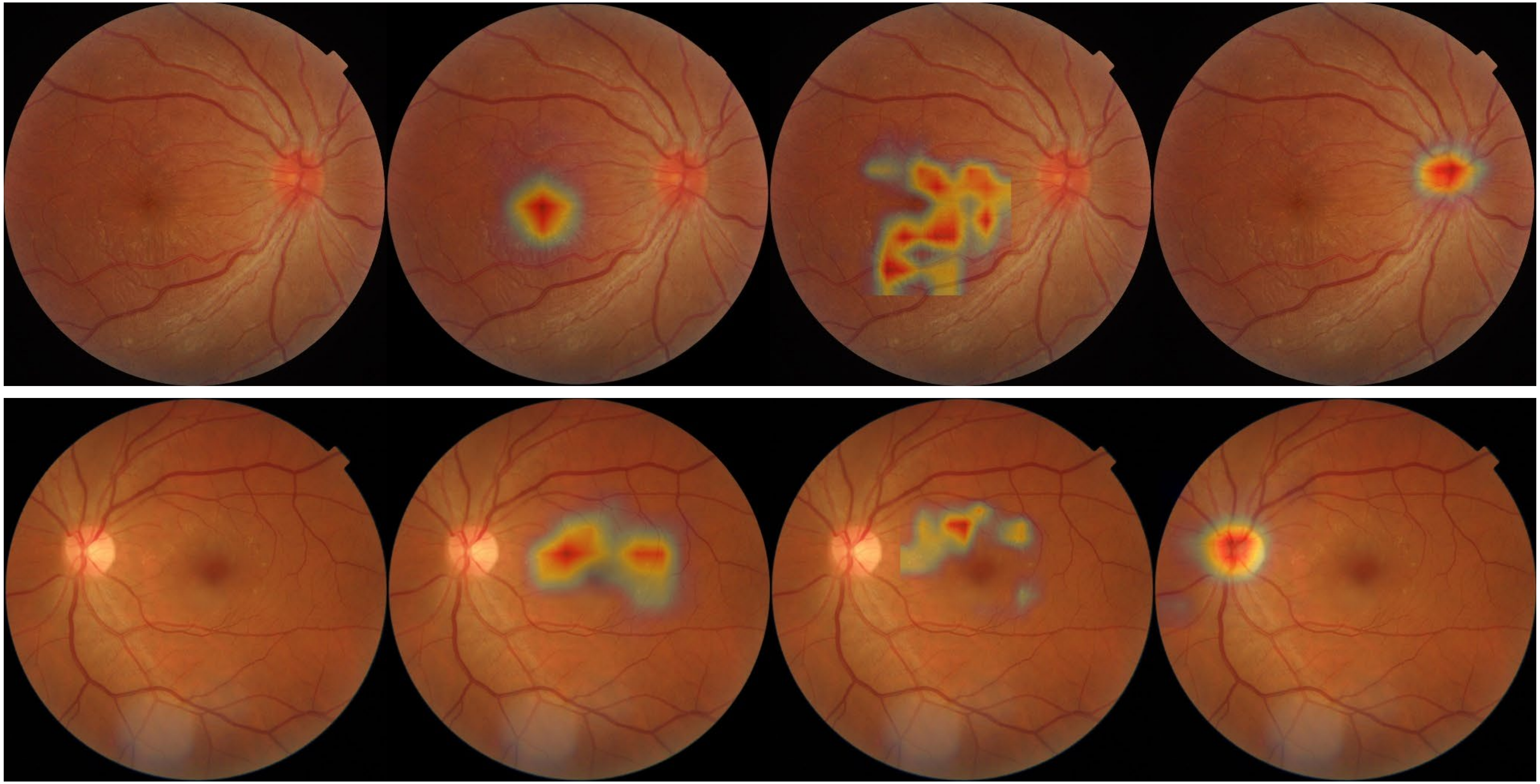

© 2022 Dong L et al. *JAMA Network Open.*

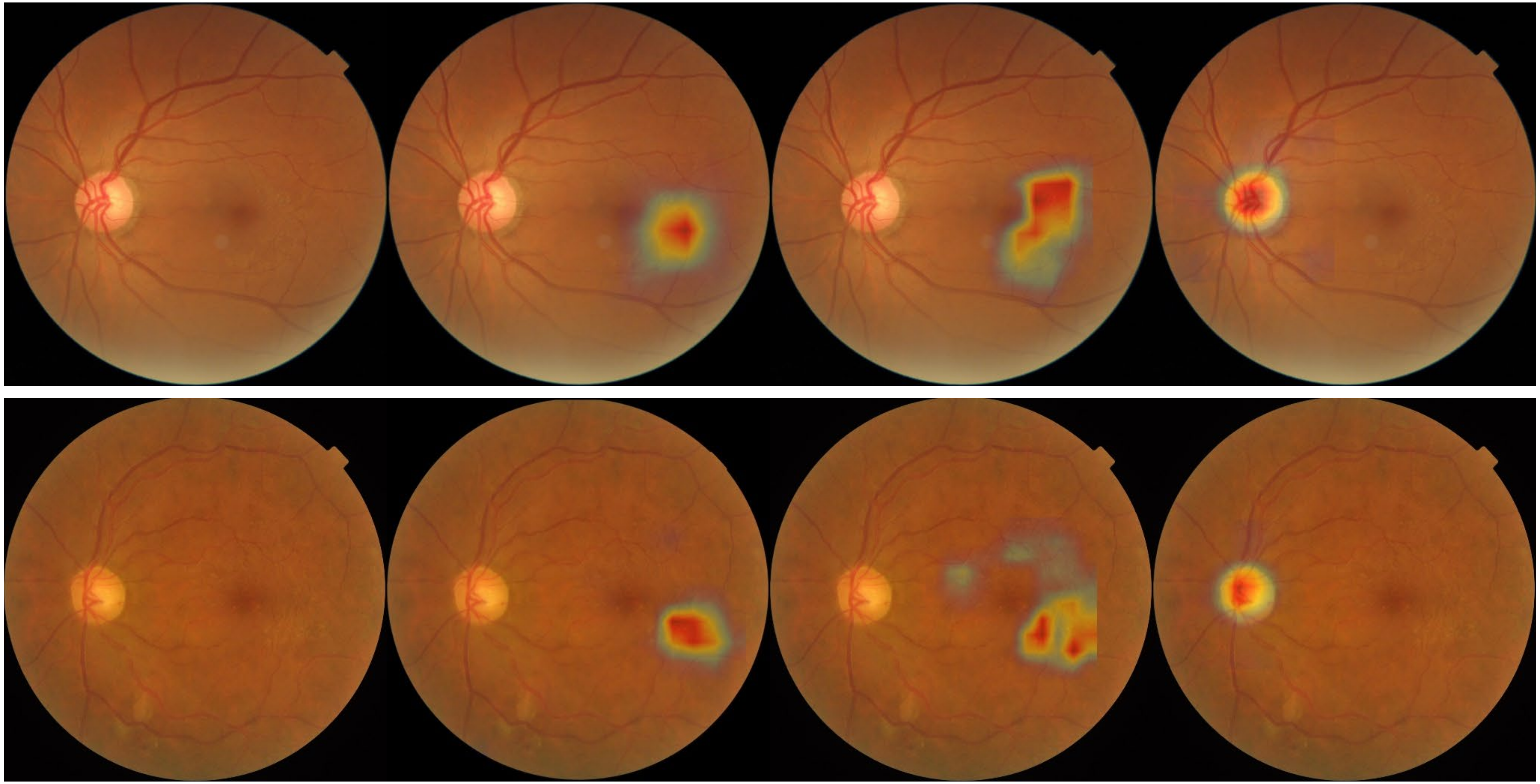

Heatmap demonstrating representative lesions, shown in original fundus image (first column), general heatmap (second column), macula heatmap (third column), and optic-disc heatmap (last column)

© 2022 Dong L et al. *JAMA Network Open.*

eFigure 11. Heatmap Visualization of Hypertensive Retinopathy

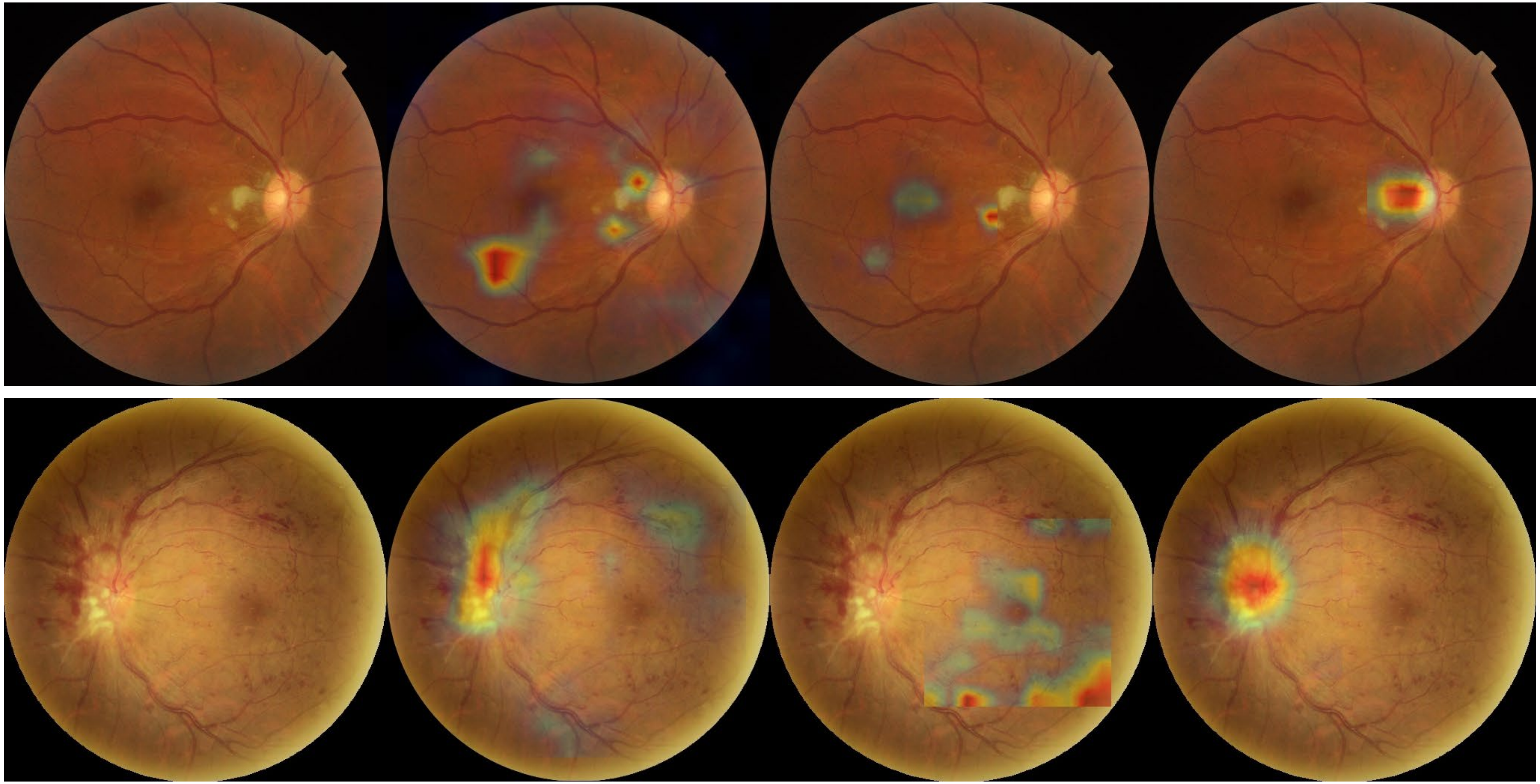

© 2022 Dong L et al. *JAMA Network Open.*

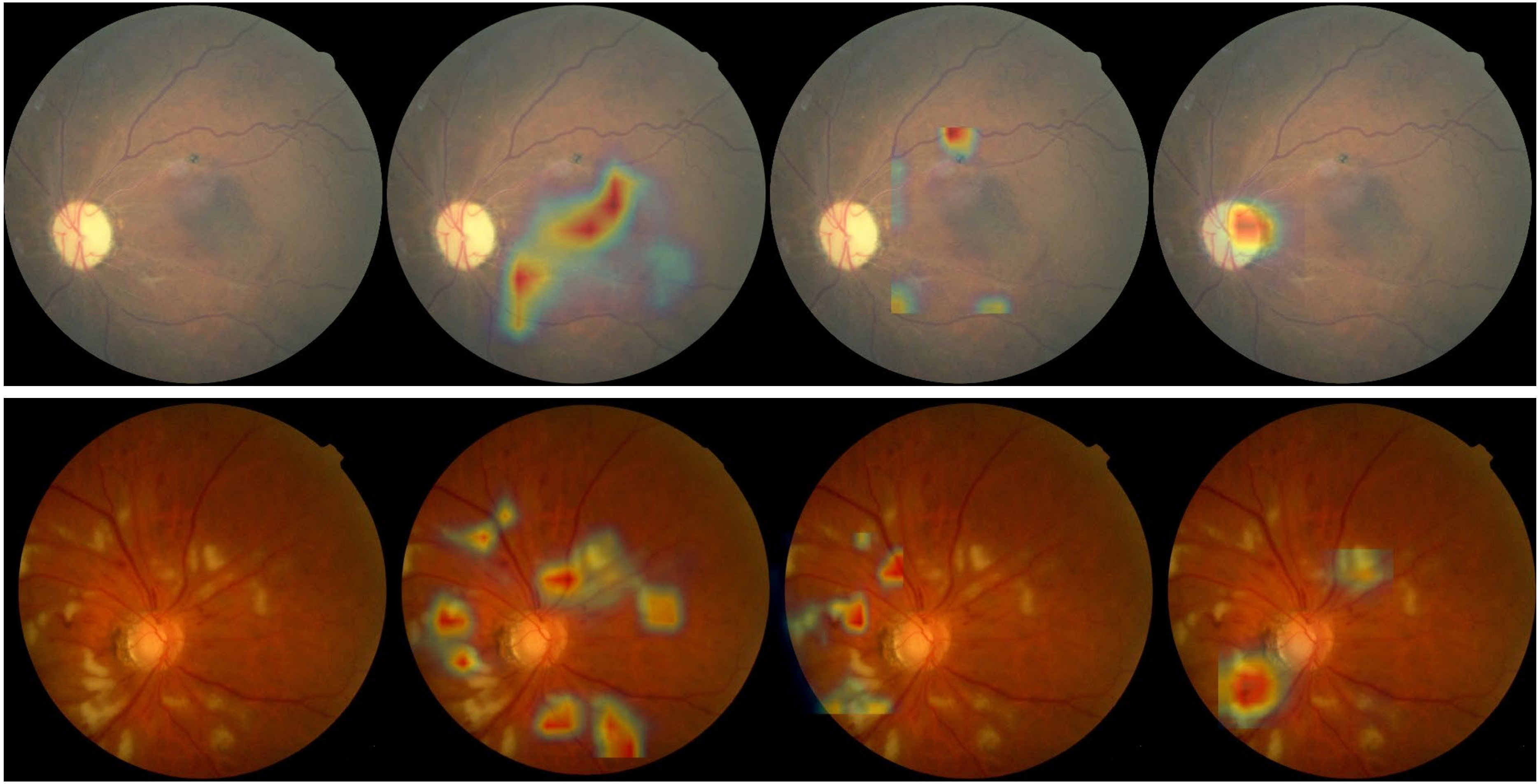

Heatmap demonstrating representative lesions, shown in original fundus image (first column), general heatmap (second column), macula heatmap (third column), and optic-disc heatmap (last column)

© 2022 Dong L et al. *JAMA Network Open.*

eFigure 12. Heatmap Visualization of Myelinated Fibers

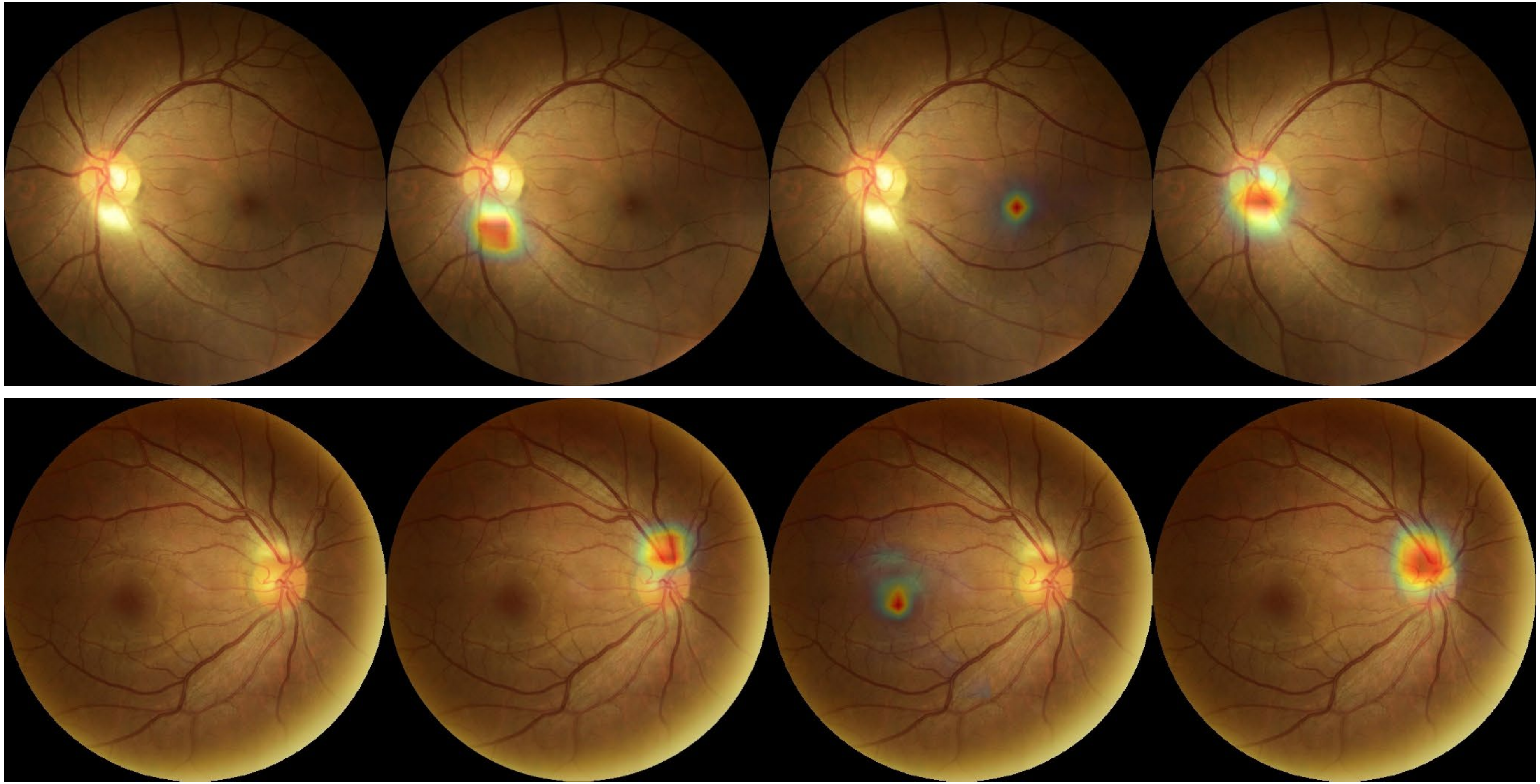

© 2022 Dong L et al. *JAMA Network Open.*

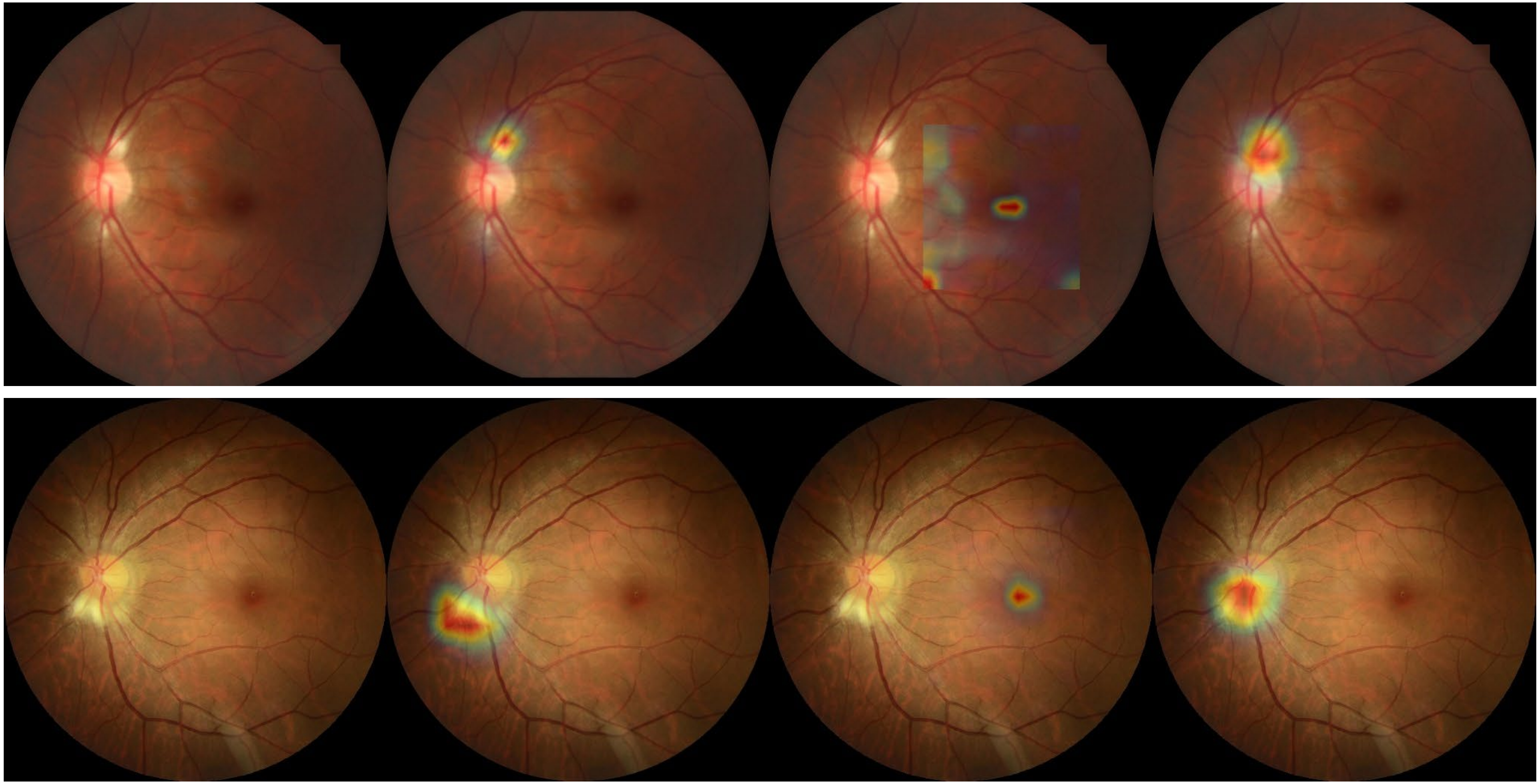

Heatmap demonstrating representative lesions, shown in original fundus image (first column), general heatmap (second column), macula heatmap (third column), and optic-disc heatmap (last column)

© 2022 Dong L et al. *JAMA Network Open.*

eFigure 13. Heatmap Visualization of Retinitis Pigmentosa

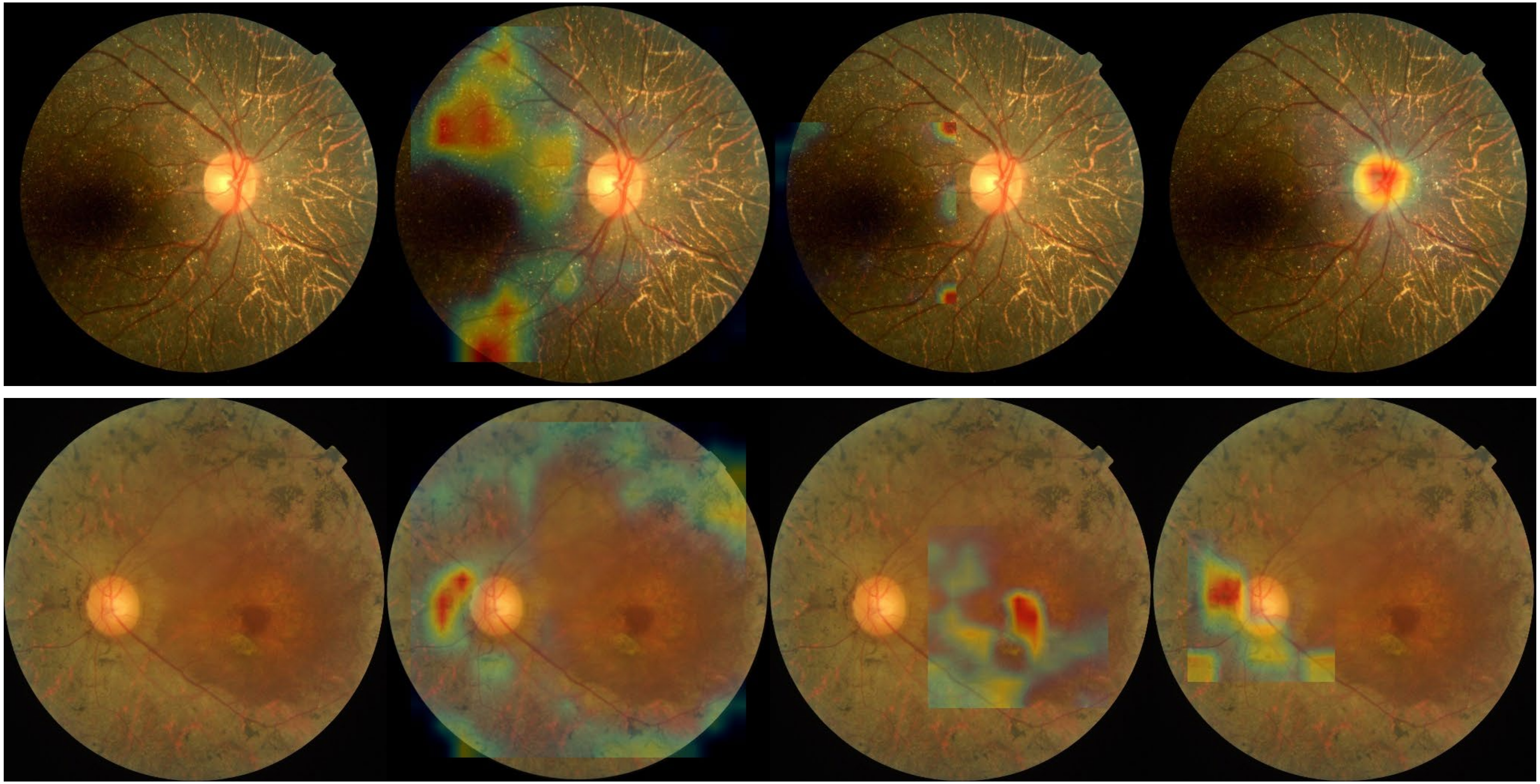

Heatmap demonstrating representative lesions, shown in original fundus image (first column), general heatmap (second column), macula heatmap (third column), and optic-disc heatmap (last column)

© 2022 Dong L et al. *JAMA Network Open.*

eFigure 14. Performance Comparison Between RAIDS and Human Ophthalmologists in Identifying Multiple Retinal Diseases

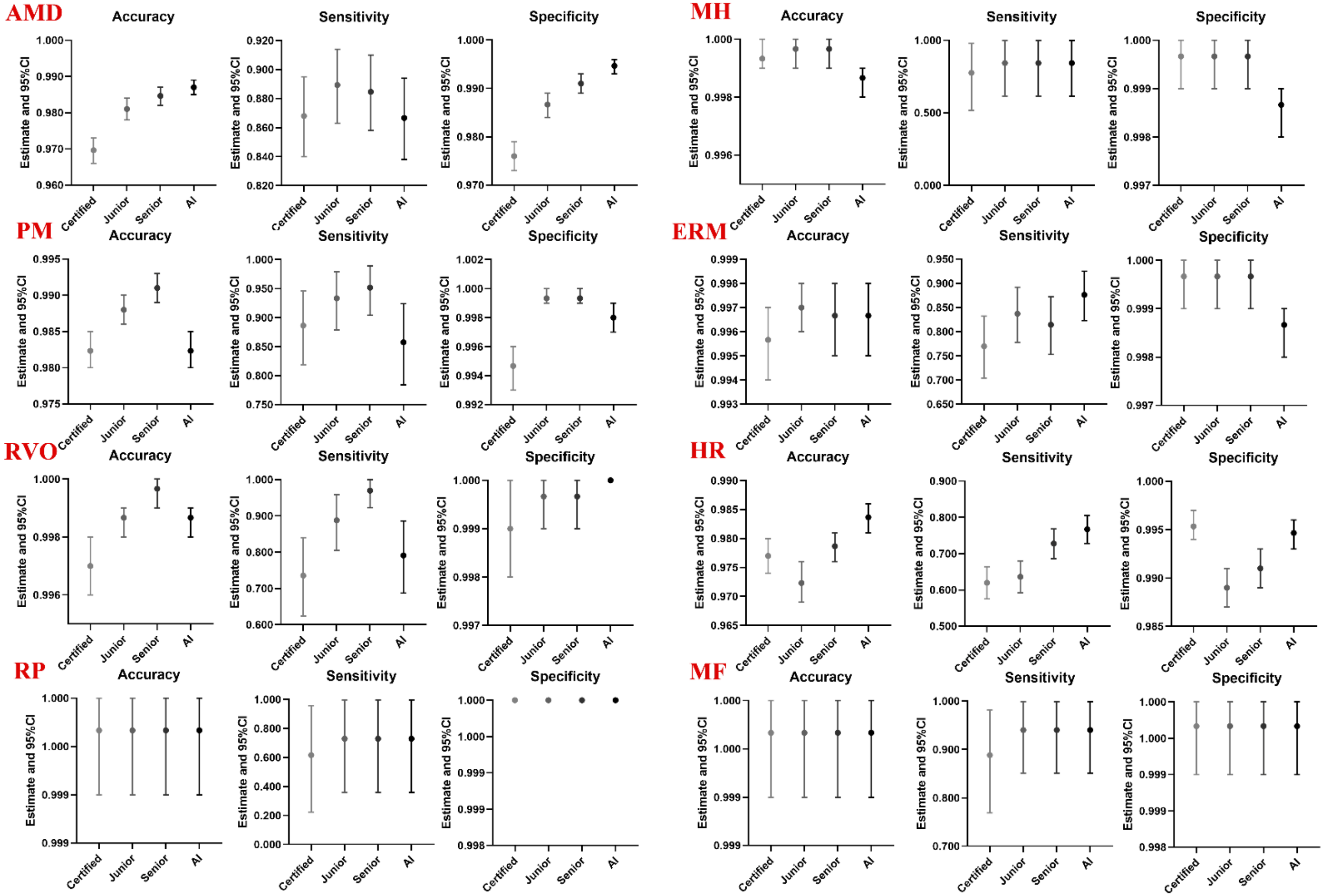

AMD, age-related macular degeneration; PM, pathologic myopia; ERM, epiretinal macular membrane; HR, hypertensive retinopathy; RVO, retinal vein occlusion; MH, macula hole; MF, myelinated fibers; RP, retinitis pigmentosa.

© 2022 Dong L et al. *JAMA Network Open.*

eFigure 15. Performance of RAIDS-Human Ophthalmologist Combination in Identifying Multiple Retinal Diseases

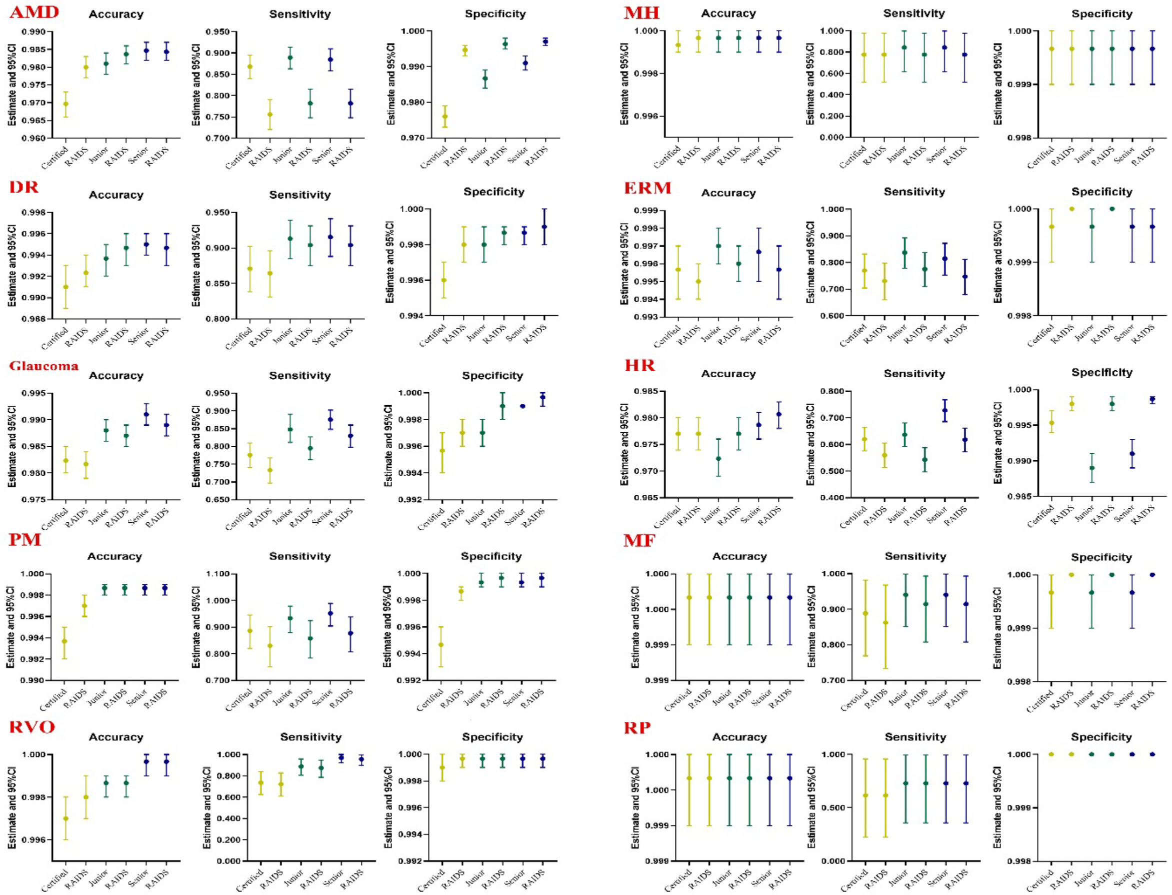

DR, referral DR; AMD, referral AMD; Glaucoma, referral possible glaucoma; PM, pathological myopia; RVO, retinal vein occlusion; MH, macula hole; ERM, epiretinal macular membrane; HR hypertensive retinopathy; MF, myelinated fibers; RP, retinitis pigmentosa.

© 2022 Dong L et al. *JAMA Network Open.*

eTable 1. Development Dataset Distributions of Retinal Diseases and Camera Manufacturers

(a)

	Training dataset	Internal validation dataset
Normal	53745	10749
Referral DR	2875	575
Referral AMD	3840	768
Referral possible glaucoma	20395	4079
Pathological myopia	2520	504
Retinal vein occlusion	270	54
Macula hole	160	32
Epiretinal macular membrane	6100	1220
Hypertensive retinopathy	12300	2460
Myelinated fibers	1580	316
Retinitis pigmentosa	185	37

(b)

Manufacturer	Images (No.)
Crystalvue	157
Nidek	448
Kowa	583
Zeiss	858
Topcon	16121
Syseye	43128
Canon	56202
Unknown	2505

© 2022 Dong L et al. *JAMA Network Open.*

eTable 2. Performance of the Retinal Artificial Intelligence Diagnosis System in the Internal Validation Dataset

	Number of cases	Accuracy	Sensitivity	Specificity
Normal	10749	0.830	0.776	0.893
Referral DR	575	0.973	0.823	0.977
Referral AMD	768	0.965	0.883	0.968
Referral possible glaucoma	4079	0.926	0.825	0.952
Pathological myopia	504	0.979	0.978	0.979
Retinal vein occlusion	54	0.959	0.963	0.959
Macula hole	32	0.989	0.938	0.989
Epiretinal macular membrane	1220	0.968	0.903	0.972
Hypertensive retinopathy	2460	0.792	0.745	0.798
Myelinated fibers	316	0.997	0.883	0.999
Retinitis pigmentosa	37	0.999	0.919	0.999

© 2022 Dong L et al. *JAMA Network Open.*

eTable 3. Performance of the Retinal Artificial Intelligence Diagnosis System (RAIDS) in Prospective Validation Dataset

(A)

**Overall**	**Number of cases**	**Accuracy [95% CI]**	**Specificity [95% CI]**	**Sensitivity [95% CI]**	**F1 score [95% CI]**
Normal	170038	0.802[0.800-0.803]	0.898[0.895-0.901]	0.780[0.778-0.782]	0.865[0.864-0.866]
Referral DR	6508	0.981[0.980-0.982]	0.986[0.985-0.986]	0.837[0.828-0.846]	0.734[0.727-0.741]
Referral AMD	7033	0.973[0.972-0.973]	0.976[0.975-0.977]	0.881[0.873-0.888]	0.685[0.678-0.691]
Referral possible glaucoma	10214	0.943[0.942-0.944]	0.950[0.949-0.951]	0.813[0.805-0.820]	0.583[0.578-0.589]
Pathological myopia	2105	0.984[0.983-0.984]	0.984[0.983-0.984]	0.971[0.964-0.978]	0.547[0.535-0.558]
Retinal vein occlusion	703	0.974[0.973-0.975]	0.974[0.973-0.975]	0.967[0.954-0.980]	0.200[0.191-0.210]
Macula hole	235	0.996[0.995-0.996]	0.996[0.995-0.996]	0.894[0.854-0.933]	0.314[0.289-0.339]
Epiretinal macular membrane	4031	0.978[0.977-0.979]	0.980[0.979-0.980]	0.891[0.881-0.901]	0.610[0.601-0.619]
Hypertensive retinopathy	8198	0.837[0.836-0.839]	0.841[0.840-0.843]	0.741[0.732-0.751]	0.264[0.260-0.268]
Myelinated fibers	812	0.999[0.999-0.999]	0.999[0.999-0.999]	0.917[0.899-0.936]	0.843[0.826-0.860]
Retinitis pigmentosa	90	0.999[0.999-1.000]	0.999[0.999-1.000]	0.933[0.882-0.985]	0.598[0.541-0.655]

CI: confidence interval, DR: diabetic retinopathy, AMD: age-related macular degeneration.

(b)

**Overall**	General-AUC	Macula-AUC	Optic-disc-AUC
Normal	0.851	0.922	0.894
Referral DR	0.983	-	-
Epiretinal macular membrane	0.987	0.975	-
Retinal vein occlusion	0.997	-	-
Referral possible glaucoma	0.950	-	0.951
Macula hole	0.991	0.991	-
Hypertensive retinopathy	0.891	-	-
Myelinated fibers	0.987	-	-
Referral AMD	0.986	0.978	-
Pathological myopia	0.996	-	-
Retinitis pigmentosa	0.997	-	-

© 2022 Dong L et al. *JAMA Network Open.*

eTable 4. Performance of the Retinal Artificial Intelligence Diagnosis System in Identifying Multiple Retinal Diseases in East China

East	Number of cases	Accuracy [95% CI]	Specificity [95% CI]	Sensitivity [95% CI]	F1 score [95% CI]
Normal	64948	0.791[0.788-0.794]	0.895[0.890-0.900]	0.768[0.765-0.771]	0.858[0.856-0.860]
Referral DR	2046	0.983[0.982-0.984]	0.987[0.986-0.987]	0.847[0.831-0.862]	0.720[0.707-0.732]
Referral AMD	2851	0.970[0.969-0.971]	0.973[0.972-0.974]	0.879[0.867-0.891]	0.677[0.667-0.688]
Referral possible glaucoma	3871	0.941[0.939-0.943]	0.948[0.947-0.950]	0.805[0.793-0.818]	0.572[0.562-0.581]
Pathological myopia	1000	0.980[0.979-0.981]	0.980[0.979-0.981]	0.973[0.963-0.983]	0.553[0.537-0.569]
Retinal vein occlusion	257	0.978[0.977-0.979]	0.978[0.977-0.979]	0.946[0.918-0.973]	0.216[0.199-0.233]
Macula hole	78	0.996[0.995-0.996]	0.996[0.996-0.996]	0.846[0.766-0.926]	0.284[0.243-0.326]
Epiretinal macular membrane	1525	0.977[0.976-0.978]	0.978[0.977-0.979]	0.906[0.892-0.921]	0.600[0.585-0.614]
Hypertensive retinopathy	2825	0.845[0.842-0.847]	0.850[0.847-0.852]	0.716[0.700-0.733]	0.247[0.241-0.254]
Myelinated fibers	321	0.999[0.998-0.999]	0.999[0.999-0.999]	0.938[0.911-0.964]	0.837[0.810-0.864]
Retinitis pigmentosa	34	0.999[0.999-1.000]	0.999[0.999-1.000]	0.971[0.914-1.000]	0.559[0.470-0.649]

CI: confidence interval, DR: diabetic retinopathy, AMD: age-related macular degeneration.

© 2022 Dong L et al. *JAMA Network Open.*

eTable 5. Performance of the Retinal Artificial Intelligence Diagnosis System in Identifying Multiple Retinal Diseases in North China

North	Number of cases	Accuracy [95% CI]	Specificity [95% CI]	Sensitivity [95% CI]	F1 score [95% CI]
Normal	55463	0.799[0.796-0.802]	0.904[0.899-0.908]	0.773[0.770-0.777]	0.860[0.858-0.863]
Referral DR	2642	0.980[0.979-0.981]	0.985[0.984-0.986]	0.836[0.822-0.850]	0.758[0.747-0.769]
Referral AMD	2308	0.972[0.971-0.974]	0.975[0.974-0.977]	0.886[0.873-0.899]	0.682[0.670-0.694]
Referral possible glaucoma	3352	0.944[0.942-0.946]	0.950[0.948-0.952]	0.824[0.811-0.837]	0.588[0.578-0.598]
Pathological myopia	449	0.988[0.987-0.989]	0.988[0.987-0.989]	0.962[0.944-0.980]	0.505[0.482-0.529]
Retinal vein occlusion	264	0.967[0.966-0.968]	0.967[0.966-0.968]	0.989[0.976-1.000]	0.186[0.172-0.201]
Macula hole	92	0.995[0.995-0.996]	0.995[0.995-0.996]	0.913[0.855-0.971]	0.340[0.298-0.382]
Epiretinal macular membrane	1380	0.976[0.975-0.977]	0.978[0.977-0.979]	0.884[0.867-0.901]	0.598[0.583-0.613]
Hypertensive retinopathy	3487	0.813[0.810-0.816]	0.815[0.812-0.818]	0.781[0.767-0.795]	0.296[0.290-0.303]
Myelinated fibers	241	0.999[0.998-0.999]	0.999[0.999-0.999]	0.892[0.853-0.931]	0.825[0.793-0.858]
Retinitis pigmentosa	16	0.999[0.999-1.000]	0.999[0.999-1.000]	0.938[0.819-1.000]	0.448[0.329-0.567]

CI: confidence interval, DR: diabetic retinopathy, AMD: age-related macular degeneration.

© 2022 Dong L et al. *JAMA Network Open.*

eTable 6. Performance of the Retinal Artificial Intelligence Diagnosis System Identifying Multiple Retinal Diseases in South China

South	Number of cases	Accuracy [95% CI]	Specificity [95% CI]	Sensitivity [95% CI]	F1 score [95% CI]
Normal	21984	0.823[0.819-0.828]	0.890[0.880-0.900]	0.812[0.807-0.818]	0.888[0.885-0.891]
Referral DR	500	0.985[0.984-0.987]	0.988[0.987-0.990]	0.834[0.801-0.867]	0.689[0.663-0.715]
Referral AMD	708	0.978[0.976-0.980]	0.980[0.979-0.982]	0.888[0.865-0.912]	0.690[0.669-0.712]
Referral possible glaucoma	1142	0.942[0.939-0.944]	0.947[0.944-0.950]	0.824[0.802-0.846]	0.557[0.540-0.574]
Pathological myopia	185	0.986[0.985-0.988]	0.986[0.985-0.988]	0.968[0.942-0.993]	0.503[0.466-0.540]
Retinal vein occlusion	43	0.983[0.981-0.985]	0.983[0.981-0.985]	0.907[0.820-0.994]	0.152[0.121-0.183]
Macula hole	25	0.996[0.995-0.996]	0.996[0.995-0.997]	0.880[0.753-1.000]	0.284[0.213-0.355]
Epiretinal macular membrane	445	0.982[0.980-0.983]	0.983[0.982-0.985]	0.892[0.863-0.921]	0.627[0.601-0.654]
Hypertensive retinopathy	534	0.883[0.879-0.887]	0.887[0.883-0.891]	0.678[0.638-0.718]	0.194[0.182-0.207]
Myelinated fibers	89	0.999[0.998-0.999]	0.999[0.999-0.999]	0.888[0.822-0.953]	0.832[0.778-0.885]
Retinitis pigmentosa	13	0.999[0.999-1.000]	1.000[0.999-1.000]	0.846[0.650-1.000]	0.629[0.468-0.789]

CI: confidence interval, DR: diabetic retinopathy, AMD: age-related macular degeneration.

© 2022 Dong L et al. *JAMA Network Open.*

eTable 7. Performance of the Retinal Artificial Intelligence Diagnosis System in Identifying Multiple Retinal Diseases in West China

West	Number of cases	Accuracy [95% CI]	Specificity [95% CI]	Sensitivity [95% CI]	F1 score [95% CI]
Normal	27643	0.815[0.811-0.819]	0.895[0.888-0.902]	0.795[0.790-0.800]	0.873[0.870-0.876]
Referral DR	1320	0.977[0.975-0.978]	0.983[0.981-0.984]	0.823[0.803-0.844]	0.728[0.712-0.744]
Referral AMD	1166	0.975[0.974-0.977]	0.979[0.978-0.981]	0.870[0.851-0.890]	0.705[0.688-0.722]
Referral possible glaucoma	1849	0.948[0.945-0.950]	0.956[0.954-0.958]	0.802[0.784-0.820]	0.620[0.606-0.634]
Pathological myopia	471	0.982[0.981-0.984]	0.982[0.981-0.984]	0.977[0.963-0.990]	0.598[0.573-0.622]
Retinal vein occlusion	139	0.973[0.971-0.974]	0.972[0.971-0.974]	0.986[0.966-1.000]	0.224[0.200-0.247]
Macula hole	40	0.996[0.995-0.996]	0.996[0.995-0.996]	0.950[0.882-1.000]	0.339[0.277-0.401]
Epiretinal macular membrane	681	0.982[0.980-0.983]	0.984[0.983-0.985]	0.871[0.846-0.896]	0.653[0.632-0.675]
Hypertensive retinopathy	1352	0.835[0.831-0.839]	0.840[0.836-0.844]	0.717[0.693-0.741]	0.253[0.243-0.263]
Myelinated fibers	161	0.999[0.999-0.999]	0.999[0.999-1.000]	0.932[0.893-0.971]	0.888[0.854-0.921]
Retinitis pigmentosa	27	1.000[0.999-1.000]	1.000[1.000-1.000]	0.926[0.827-1.000]	0.820[0.723-0.916]

CI: confidence interval, DR: diabetic retinopathy, AMD: age-related macular degeneration.

© 2022 Dong L et al. *JAMA Network Open.*

eTable 8. Baseline Characteristics of Participants in the Reader Study

	Beijing Eye study (n=5084)	Kailuan Eye study (n=5000)	Total (n=10084)
Number of participants	1624	1918	3542
Mean age (range)/years	62 (50-89)	48 (20-85)	52 (20-89)
Female (%)	55.3%	37.0%	45.4%
Normal	3991	3719	7710
Referral DR	192	255	447
Referral AMD	219	385	604
Referral possible glaucoma	322	286	608
Pathological myopia	61	43	104
Retinal vein occlusion	34	36	70
Macula hole	6	6	12
Epiretinal macular membrane	89	87	176
Hypertensive retinopathy	232	247	479
Myelinated fibers	25	10	35
Retinitis pigmentosa	0	6	6

© 2022 Dong L et al. *JAMA Network Open.*

eTable 9. Performance of Human Ophthalmologists in Identifying Multiple Retinal Diseases in the Reader Study

	Number of cases	Human ophthalmologists	Accuracy [95% CI]	Specificity [95% CI]	Sensitivity [95% CI]
Normal	7710	Certified ophthalmologists	0.930 [0.924-0.935]	0.837 [0.821-0.851]	0.958 [0.954-0.963]
		Junior retinal specialists	0.945 [0.940-0.949]	0.864 [0.849-0.877]	0.970 [0.965-0.973]
		Senior retinal specialists	0.945 [0.940-0.949]	0.885 [0.871-0.898]	0.978 [0.975-0.982]
		RAIDS	0.953 [0.949-0.957]	0.917 [0.906-0.928]	0.964 [0.960-0.968]
Referral DR	447	Certified ophthalmologists	0.991 [0.989-0.993]	0.996 [0.995-0.997]	0.872 [0.838-0.902]
		Junior retinal specialists	0.994 [0.992-0.995]	0.998 [0.997-0.999]	0.915 [0.885-0.939]
		Senior retinal specialists	0.995 [0.994-0.996]	0.999 [0.998-0.999]	0.917 [0.888-0.941]
		RAIDS	0.985 [0.982-0.987]	0.985 [0.983-0.987]	0.973 [0.954-0.986]
Referral AMD	604	Certified ophthalmologists	0.970 [0.966-0.973]	0.976 [0.973-0.979]	0.869 [0.840-0.895]
		Junior retinal specialists	0.981 [0.978-0.984]	0.987 [0.984-0.989]	0.891 [0.863-0.914]
		Senior retinal specialists	0.985 [0.982-0.987]	0.991 [0.989-0.993]	0.886 [0.858-0.910]
		RAIDS	0.987 [0.985-0.989]	0.995 [0.993-0.996]	0.868 [0.838-0.894]
Referral possible glaucoma	608	Certified ophthalmologists	0.982 [0.980-0.985]	0.996 [0.994-0.997]	0.776 [0.741-0.809]
		Junior retinal specialists	0.988 [0.986-0.990]	0.997 [0.996-0.998]	0.842 [0.811-0.890]
		Senior retinal specialists	0.991 [0.989-0.993]	0.999 [0.999-0.999]	0.877 [0.848-0.902]
		RAIDS	0.982 [0.980-0.985]	0.985 [0.982-0.987]	0.942 [0.921-0.960]

N=10,084

© 2022 Dong L et al. *JAMA Network Open.*

	Number of cases	Human ophthalmologists	Accuracy	Specificity	Sensitivity
Pathological myopia	104	Certified ophthalmologists	0.994 [0.992-0.995]	0.995 [0.993-0.996]	0.894 [0.819-0.946]
		Junior retinal specialists	0.999 [0.998-0.999]	0.999 [0.999-1.000]	0.942 [0.879-0.979]
		Senior retinal specialists	0.999 [0.998-0.999]	0.999 [0.999-1.000]	0.962 [0.904-0.989]
		RAIDS	0.996 [0.995-0.998]	0.998 [0.997-0.999]	0.865 [0.784-0.924]
Retinal vein occlusion	70	Certified ophthalmologists	0.997 [0.996-0.998]	0.999 [0.998-1.000]	0.743 [0.624-0.840]
		Junior retinal specialists	0.999 [0.998-0.999]	1.000 [0.999-1.000]	0.900 [0.805-0.959]
		Senior retinal specialists	1.000 [0.999-1.000]	1.000 [0.999-1.000]	0.986 [0.923-1.000]
		RAIDS	0.999 [0.998-0.999]	1.000 [1.000-1.000]	0.800 [0.687-0.886]
Macula hole	12	Certified ophthalmologists	0.999 [0.999-1.000]	1.000 [0.999-1.000]	0.833 [0.516-0.979]
		Junior retinal specialists	1.000 [0.999-1.000]	1.000 [0.999-1.000]	0.917 [0.615-0.998]
		Senior retinal specialists	1.000 [0.999-1.000]	1.000 [0.999-1.000]	0.917 [0.615-0.998]
		RAIDS	0.999 [0.998-0.999]	0.999 [0.998-0.999]	0.917 [0.615-0.998]
Epiretinal macular membrane	176	Certified ophthalmologists	0.996 [0.994-0.997]	1.000 [0.999-1.000]	0.773 [0.704-0.832]
		Junior retinal specialists	0.997 [0.996-0.998]	1.000 [0.999-1.000]	0.841 [0.778-0.892]
		Senior retinal specialists	0.997 [0.995-0.998]	1.000 [0.999-1.000]	0.818 [0.753-0.872]
		RAIDS	0.997 [0.995-0.998]	0.999 [0.998-0.999]	0.881 [0.823-0.925]
Hypertensive retinopathy	479	Certified ophthalmologists	0.977 [0.974-0.980]	0.995 [0.994-0.997]	0.620 [0.575-0.664]
		Junior retinal specialists	0.972 [0.969-0.976]	0.989 [0.987-0.991]	0.637 [0.592-0.680]
		Senior retinal specialists	0.979 [0.976-0.981]	0.991 [0.989-0.993]	0.729 [0.686-0.768]
		RAIDS	0.984 [0.981-0.986]	0.995 [0.993-0.996]	0.768 [0.728-0.805]

© 2022 Dong L et al. *JAMA Network Open.*

	Number of cases	Human ophthalmologists	Accuracy	Specificity	Sensitivity
Myelinated fibers	35	Certified ophthalmologists	1.000 [0.999-1.000]	1.000 [0.999-1.000]	0.914 [0.769-0.982]
		Junior retinal specialists	1.000 [0.999-1.000]	1.000 [0.999-1.000]	0.971 [0.851-0.999]
		Senior retinal specialists	1.000 [0.999-1.000]	1.000 [0.999-1.000]	0.971 [0.851-0.999]
		RAIDS	1.000 [0.999-1.000]	1.000 [0.999-1.000]	0.971 [0.851-0.999]
Retinitis pigmentosa	6	Certified ophthalmologists	1.000 [0.999-1.000]	1.000 [1.000-1.000]	0.667 [0.223-0.957]
		Junior retinal specialists	1.000 [0.999-1.000]	1.000 [1.000-1.000]	0.833 [0.359-0.996]
		Senior retinal specialists	1.000 [0.999-1.000]	1.000 [1.000-1.000]	0.833 [0.359-0.996]
		RAIDS	1.000 [0.999-1.000]	1.000 [1.000-1.000]	0.833 [0.359-0.996]

RAIDS: Retinal Artificial Intelligence Diagnosis System, CI: confidence interval, DR: diabetic retinopathy, AMD: age-related macular degeneration.

© 2022 Dong L et al. *JAMA Network Open.*

eTable 10. K Scores Between Retinal Artificial Intelligence Diagnosis System and Different Groups of Human Experts in the Reader Study

Abnormalities	Certified ophthalmologist	Junior retinal specialist	Senior retinal specialist
Normal	0.7221	0.7390	0.7635
Referral DR	0.6463	0.7173	0.7351
Referral AMD	0.6463	0.7173	0.7351
Referral possible glaucoma	0.7397	0.7670	0.7906
Pathological myopia	0.6598	0.7904	0.8015
Retinal vein occlusion	0.7572	0.8215	0.8518
Macula hole	0.5255	0.5255	0.5548
Epiretinal macular membrane	0.8103	0.8147	0.7930
Hypertensive retinopathy	0.6501	0.5473	0.6095
Myelinated fibers	0.8728	0.8915	0.8915
Retinitis pigmentosa	0.8888	0.7999	0.7999
Average	0.7199	0.7392	0.7569

DR: diabetic retinopathy, AMD: age-related macular degeneration.

© 2022 Dong L et al. *JAMA Network Open.*

eTable 11. Intergroup Correlation Between Ophthalmologists.

Group 1	Group 2	Kappa score
Certified ophthalmologists	Junior retinal specialists	0.794
Certified ophthalmologists	Senior retinal specialists	0.769
Junior retinal specialists	Senior retinal specialists	0.912

© 2022 Dong L et al. *JAMA Network Open.*

eTable 12. Efficiency Analysis of the DL Algorithm vs Human Ophthalmologists in Identifying Multiple Retinal Diseases in the Reader Study

	Time cost per photograph (second)
RAIDS	0.30
Certified ophthalmologists	10.88
Junior retinal specialists	8.68
Senior retinal specialists	7.68
RAIDS + certified ophthalmologists†	2.95
RAIDS + junior retinal specialists†	2.40
RAIDS + senior retinal specialists†	2.15

† DL algorithm screened all images to filter the normal images as the first reader, and then human ophthalmologists only diagnosed the remaining images as the second reader

© 2022 Dong L et al. *JAMA Network Open.*

eTable 13. Performance of the Combination of Certified Ophthalmologists and the Retinal Artificial Intelligence Diagnosis System in the Reader Study

	Number of cases	Accuracy (95% CI)	Specificity [95% CI]	Sensitivity [95% CI]
Referral DR	447	0.992 [0.991-0.994]	0.998 [0.997-0.999]	0.866 [0.831-0.896]
Referral AMD	604	0.980 [0.977-0.983]	0.995 [0.993-0.996]	0.757 [0.720-0.790]
Referral possible glaucoma	608	0.982 [0.979-0.984]	0.997 [0.996-0.998]	0.734 [0.697-0.768]
Pathological myopia	104	0.997 [0.996-0.998]	0.999 [0.998-0.999]	0.837 [0.751-0.902]
Retinal vein occlusion	70	0.998 [0.997-0.999]	1.000 [0.999-1.000]	0.729 [0.609-0.828]
Macula hole	12	1.000 [0.999-1.000]	1.000 [0.999-1.000]	0.833 [0.516-0.979]
Epiretinal macular membrane	176	0.995 [0.994-0.996]	1.000 [1.000-1.000]	0.733 [0.661-0.797]
Hypertensive retinopathy	479	0.977 [0.974-0.980]	0.998 [0.997-0.999]	0.559 [0.514-0.605]
Myelinated fibers	35	1.000 [0.999-1.000]	1.000 [1.000-1.000]	0.886 [0.733-0.968]
Retinitis pigmentosa	6	1.000 [0.999-1.000]	1.000 [1.000-1.000]	0.667 [0.223-0.957]

CI: confidence interval, DR: diabetic retinopathy, AMD: age-related macular degeneration.

© 2022 Dong L et al. *JAMA Network Open.*

eTable 14. Performance of the Combination of Junior Retinal Specialists and the Retinal Artificial Intelligence Diagnosis System in the Reader Study

	Number of cases	Accuracy (95% CI)	Specificity [95% CI]	Sensitivity [95% CI]
Referral DR	447	0.995 [0.993-0.996]	0.999 [0.998-0.999]	0.906 [0.875-0.931]
Referral AMD	604	0.984 [0.981-0.986]	0.996 [0.995-0.998]	0.783 [0.748-0.815]
Referral possible glaucoma	608	0.987 [0.985-0.989]	0.999 [0.998-1.000]	0.796 [0.762-0.827]
Pathological myopia	104	0.999 [0.998-0.999]	1.000 [0.999-1.000]	0.865 [0.784-0.924]
Retinal vein occlusion	70	0.999 [0.998-0.999]	1.000 [0.999-1.000]	0.886 [0.787-0.949]
Macula hole	12	1.000 [0.999-1.000]	1.000 [0.999-1.000]	0.833 [0.516-0.979]
Epiretinal macular membrane	176	0.996 [0.995-0.997]	1.000 [1.000-1.000]	0.778 [0.710-0.837]
Hypertensive retinopathy	479	0.977 [0.974-0.980]	0.998 [0.997-0.999]	0.543 [0.497-0.588]
Myelinated fibers	35	1.000 [0.999-1.000]	1.000 [1.000-1.000]	0.943 [0.808-0.993]
Retinitis pigmentosa	6	1.000 [0.999-1.000]	1.000 [1.000-1.000]	0.833 [0.359-0.996]

CI: confidence interval, DR: diabetic retinopathy, AMD: age-related macular degeneration.

© 2022 Dong L et al. *JAMA Network Open.*

eTable 15. Performance of the Combination of Senior Retinal Specialists and the Retinal Artificial Intelligence Diagnosis System in the Reader Study

	Number of cases	Accuracy (95% CI)	Specificity [95% CI]	Sensitivity [95% CI]
Referral DR	447	0.995 [0.993-0.996]	0.999 [0.998-1.000]	0.906 [0.875-0.931]
Referral AMD	604	0.984 [0.982-0.987]	0.997 [0.996-0.998]	0.783 [0.748-0.815]
Referral possible glaucoma	608	0.989 [0.987-0.991]	1.000 [0.999-1.000]	0.831 [0.798-0.860]
Pathological myopia	104	0.999 [0.998-0.999]	1.000 [0.999-1.000]	0.885 [0.807-0.939]
Retinal vein occlusion	70	1.000 [0.999-1.000]	1.000 [0.999-1.000]	0.971 [0.901-0.997]
Macula hole	12	1.000 [0.999-1.000]	1.000 [0.999-1.000]	0.833 [0.516-0.979]
Epiretinal macular membrane	176	0.996 [0.994-0.997]	1.000 [0.999-1.000]	0.750 [0.679-0.812]
Hypertensive retinopathy	479	0.981 [0.978-0.983]	0.999 [0.998-0.999]	0.618 [0.573-0.662]
Myelinated fibers	35	1.000 [0.999-1.000]	1.000 [1.000-1.000]	0.943 [0.808-0.993]
Retinitis pigmentosa	6	1.000 [0.999-1.000]	1.000 [1.000-1.000]	0.833 [0.359-0.996]

CI: confidence interval, DR: diabetic retinopathy, AMD: age-related macular degeneration.

© 2022 Dong L et al. *JAMA Network Open.*
